# Supplementary material for: Spectra stable blue perovskite light-emitting diodes
Source: Nat Commun. 2019 Apr 23;10:1868. doi: 10.1038/s41467-019-09794-7 (PMC6478869; doi:10.1038/s41467-019-09794-7)
Supplement: Supplementary file 1 — Supplementary Information [file 41467_2019_9794_MOESM1_ESM.pdf]

Supplementary Information

Spectra stable blue perovskite light-emitting diodes

Jiang *et al.*

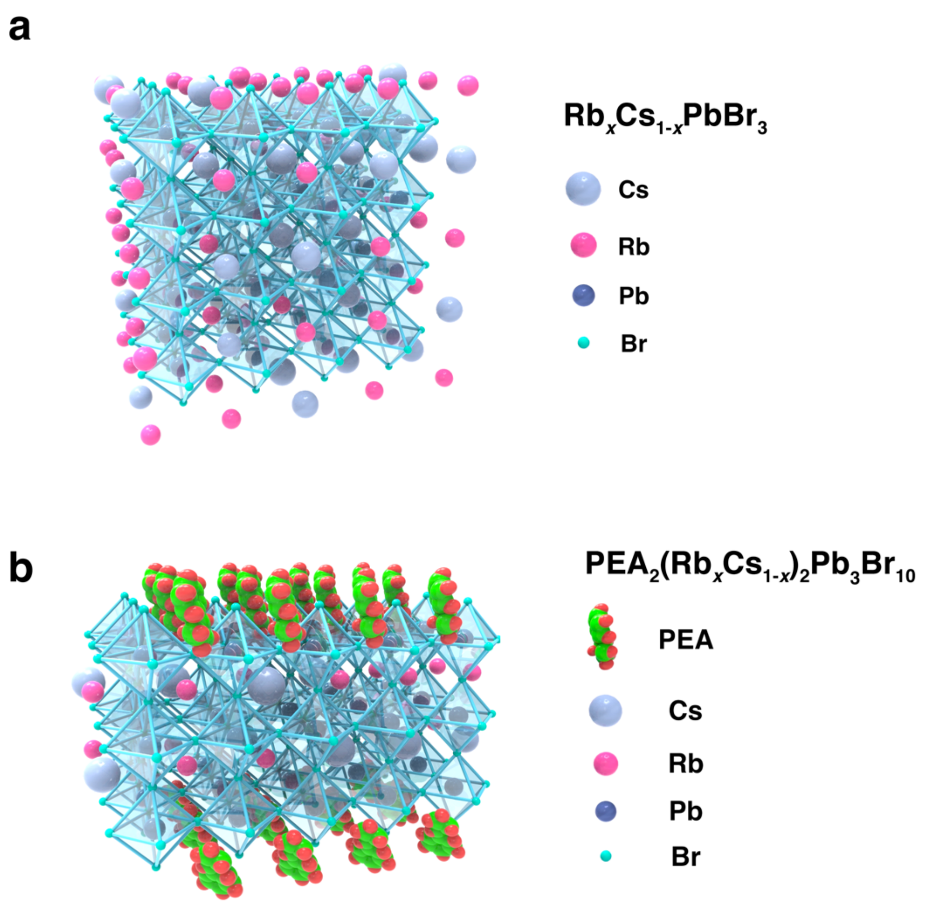

Supplementary Figure 1 | Cartoon structure of Rb-Cs alloyed perovskites. The crystalline structure diagrams of **(a)** 3D perovskites $Rb_xCs_{1-x}PbBr_3$ and **(b)** quasi-2D perovskites $PEA_2(Rb_xCs_{1-x})_2Pb_3Br_{10}$.

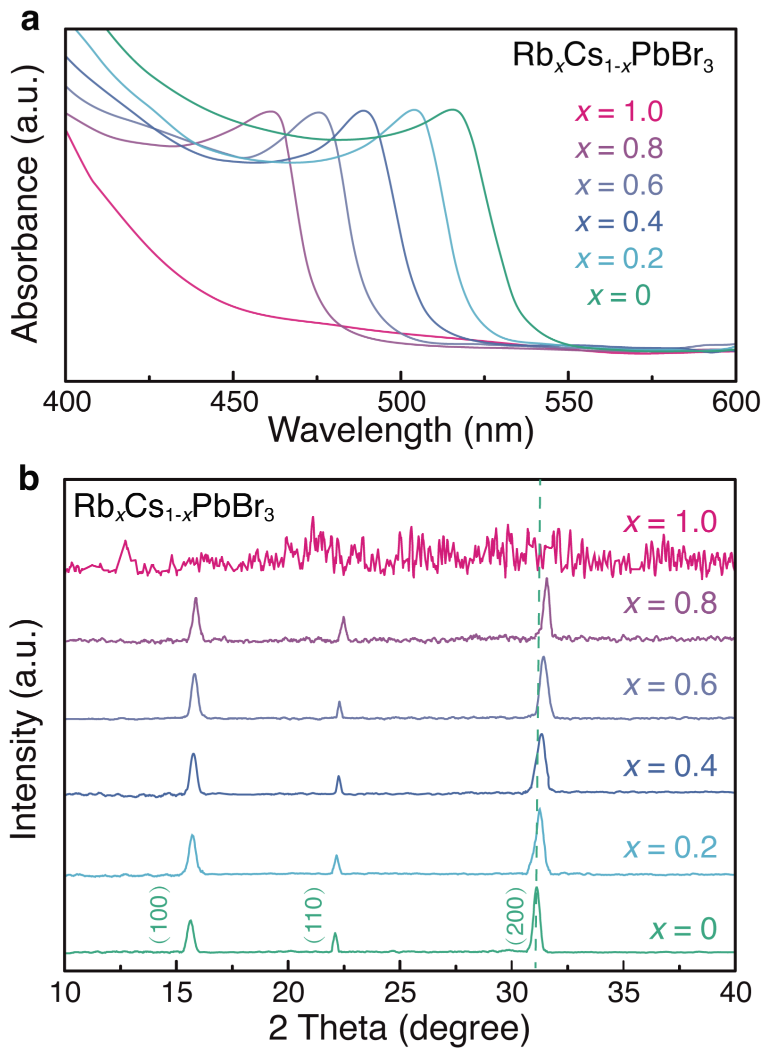

Supplementary Figure 2 | UV-vis absorption spectra and X-ray diffraction patterns of films. (a) UV-vis absorption spectra and **(b)** XRD patterns of 3D perovskites $Rb_xCs_{1-x}PbBr_3$ with different values of 'x'. Optical bandgaps of the $Rb_xCs_{1-x}PbBr_3$ films increase from 2.31 eV to 2.60 eV ($0 \leq x \leq 0.8$) with increasing ratio of Rb to Cs (Supplementary Figure 29). Meanwhile, XRD peaks are also shifted to higher angles. These phenomena could convincingly demonstrate the miscibility of Rb-based and Cs-based perovskite[1]. Unfortunately, the resulting perovskites' stability significantly decreases as the ratio of Rb to Cs increasing. Especially, the pure-Rb perovskite ($RbPbBr_3$) exhibits an amorphous state as soon as prepared. The decreased stability could be ascribed to the increased octahedral tilting distortion causing by continuous Rb^+ substitution[1].

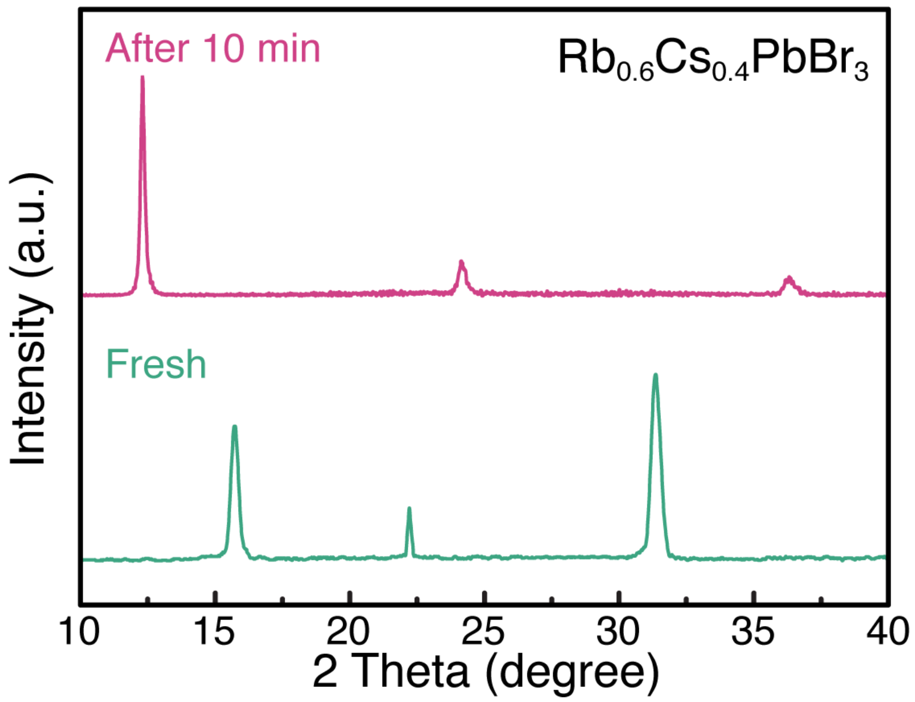

Supplementary Figure 3 | XRD patterns of perovskite films. XRD patterns of 3D perovskite $Rb_{0.6}Cs_{0.4}PbBr_3$ film before and after ageing under ambient condition. Though the 3D perovskite $Rb_{0.6}Cs_{0.4}PbBr_3$ exhibits an orthorhombic perovskite phase, it could be completely decomposed within several minutes. Thus, the poor crystal stability is a big obstacle for Rb-Cs alloyed 3D perovskite application.

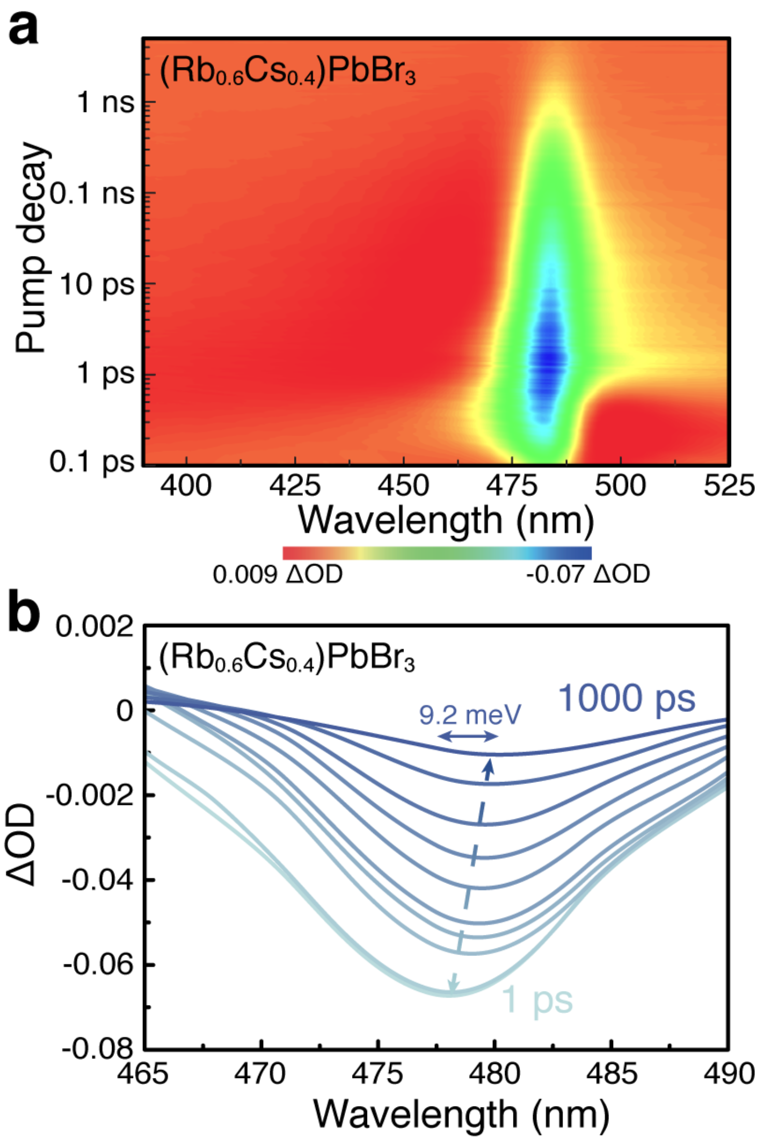

Supplementary Figure 4 | TA measurement for 3D perovskite $Rb_{0.6}Cs_{0.4}PbBr_3$. (a) Time-wavelength dependent TA color map and **(b)** TA spectra at selected timescales for $Rb_{0.6}Cs_{0.4}PbBr_3$. As shown, the bleaching edge of 3D $Rb_{0.6}Cs_{0.4}PbBr_3$ perovskite is around 495 nm, which is consistent with the result from UV-vis absorption spectra (Supplementary Figure 2). Furthermore, we also carried out the band-tail measurement from the transient bleaching, and found an around 9.2 meV Urbach tail in it, demonstrating a largely energy disorder derived from serious defect state in the film[2]. Thus, these defect state could result in significant non-radiative recombination and extremely low PLQY and PL lifetime (Fig. 3c)[3].

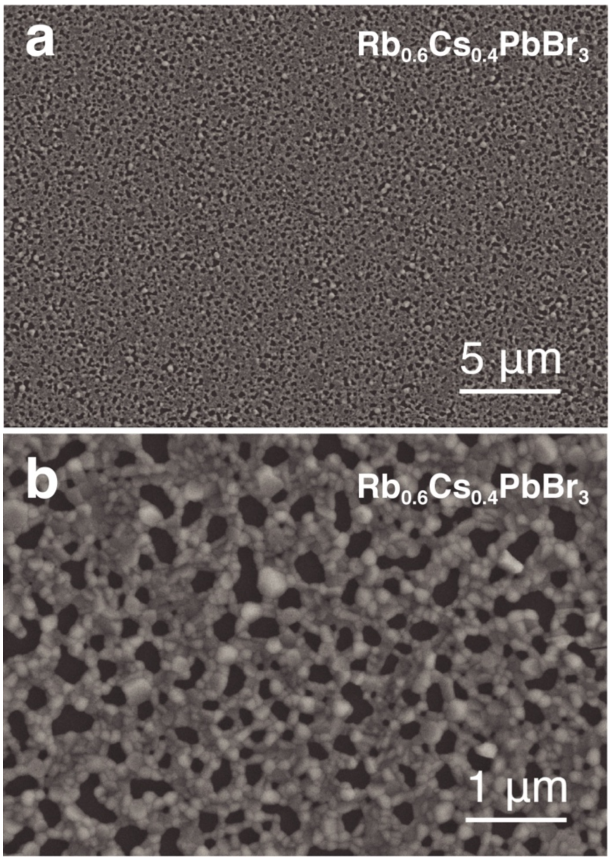

Supplementary Figure 5 | Morphology of perovskite film. Top-view SEM images of 3D $Rb_{0.6}Cs_{0.4}PbBr_3$ with a scale bar of **(a)** 5 µm and **(b)** 1 µm. The 3D perovskite $Rb_{0.6}Cs_{0.4}PbBr_3$ shows a rough and uneven film with poor surface coverage, which will lead to serious leakage current in the devices.

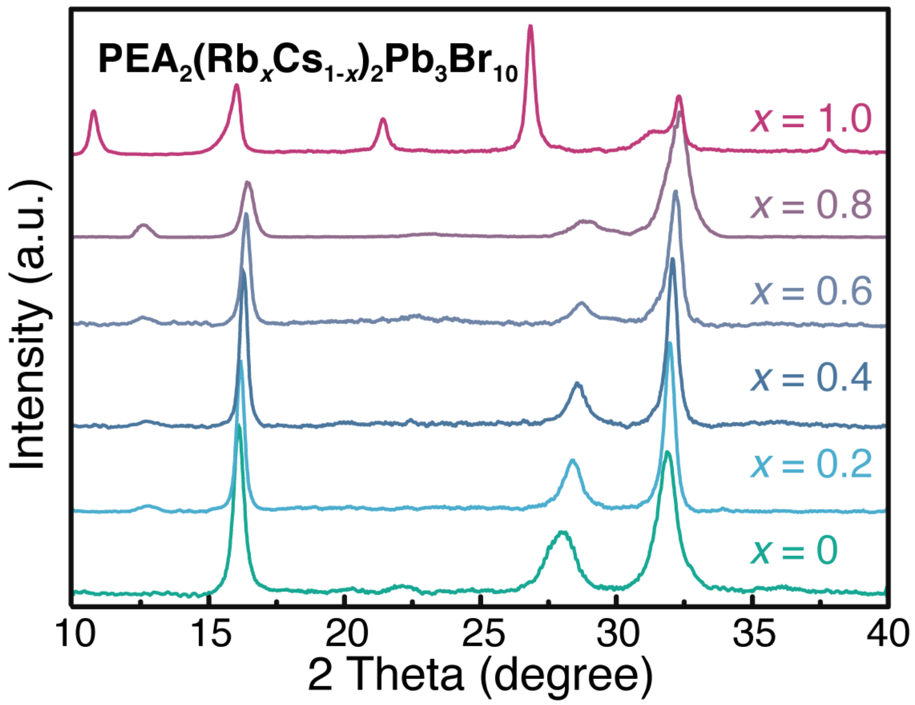

Supplementary Figure 6 | XRD patterns of the perovskite films. XRD patterns of $PEA_2(Rb_xCs_{1-x})_2Pb_3Br_{10}$ with different ratio of Rb to Cs ($0 \leq x \leq 1$). As shown, XRD peaks are shifted to higher angles with increasing amount of RbBr ($0 \leq x \leq 0.8$), demonstrating the miscibility of Rb-based and Cs-based quasi-2D perovskite. Due to the serious octahedral distortion in pure Rb-based quasi-2D perovskite ($x = 1$), a mass of $n = 1$ species could be found in the XRD pattern.

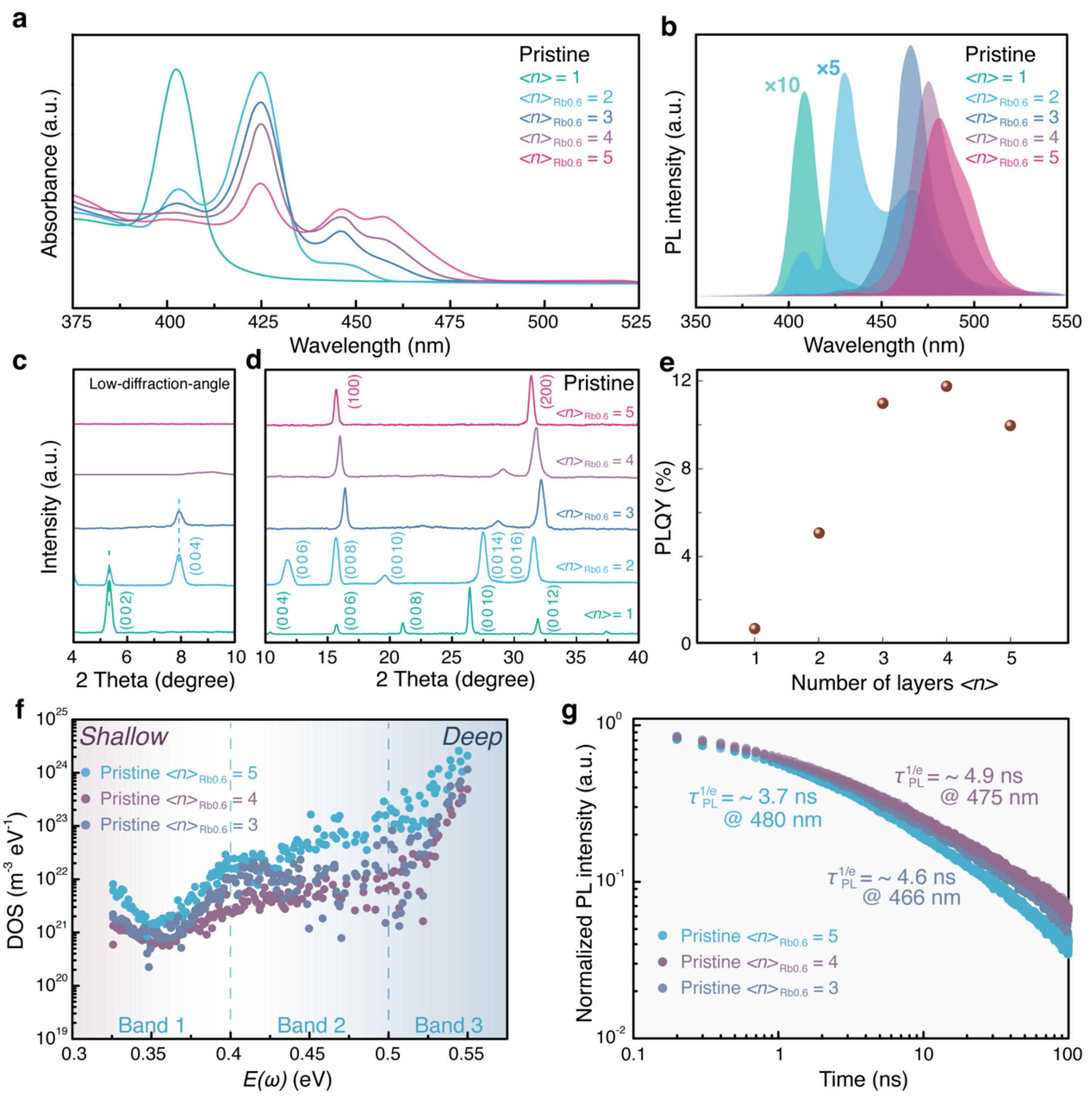

Supplementary Figure 7 | Characteristics of quasi-2D perovskite films. (a) UV-vis absorption and **(b)** steady-state PL spectra; **(c)** low- and **(d)** wide-diffraction-angle XRD patterns; and **(e)** PLQYs of quasi-2D $PEA_2(Rb_{0.6}Cs_{0.4})_{n-1}Pb_nBr_{3n+1}$ perovskite with different <*n*>-values ($1 \leq n \leq 5$). **(f)** TAS analysis of trap density of states (*t*DOS), and **(g)** PL lifetime for pristine $<n>_{Rb0.6}$ = 3, 4 and 5 perovskites.

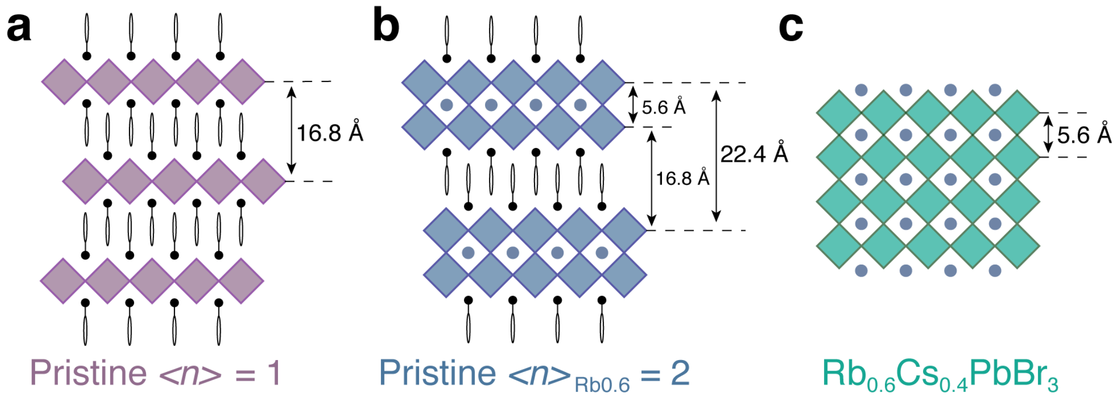

Supplementary Figure 8 | Simplified schematic representation of the crystal structures of $PEA_2(Rb_{0.6}Cs_{0.4})_{n-1}Pb_nPb_{3n+1}$ (*n* = 1, 2 and ∞). The lattice/unit constants are calculated from Supplementary Figure 7c, d by Bragg diffraction equation, and the detailed parameters are shown in Supplementary Table 2[4]. As shown, the (0 0 k) diffraction peaks were observed in <*n*> = 1 perovskite with a layer lattice constant (*d*) of 16.8 Å, indicating the formation of 2D (*n* = 1 species) perovskite[5]. Furthermore, it can also be calculated that the *d* of 3D perovskite $Rb_{0.6}Cs_{0.4}PbBr_3$ is around 5.6 Å from the diffraction peak of (1 0 0) and (2 0 0). As expected, the *d* of <*n*> = 2 is measured to be 22.4 Å, which fully fits the expected *n* = 2 quasi-2D layered perovskite structure (16.8 Å + (*n* - 1) × 5.6 Å, *n* = 2).

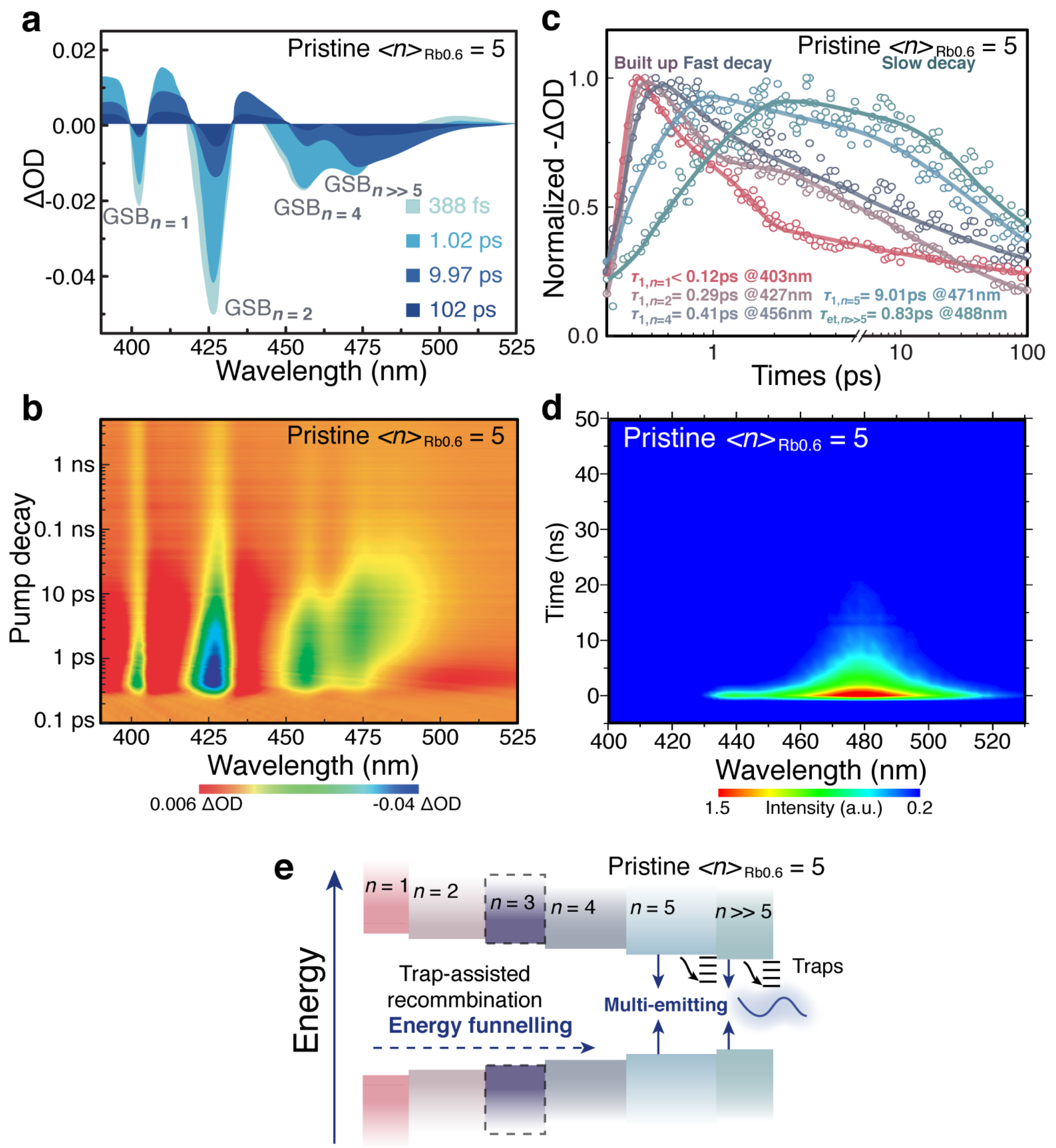

Supplementary Figure 9 | Photocarrier transportation and recombination dynamics in pristine $<n>_{\mathrm{Rb0.6}}$ = 5. **(a)** TA spectra at selected timescales, **(b)** time-wavelength dependent TA color map and **(c)** TA spectra at different wavelength as a function of delay time for pristine $<n>_{\mathrm{Rb0.6}}$ = 5 perovskite. **(d)** Time-resolved photoluminescence (TRPL) color map of pristine $<n>_{\mathrm{Rb0.6}}$ = 5 perovskite film. The excitation fluence used was 105.8 nJ cm^{-2}. **(e)** Schematic representation of energy transfer process through pristine $<n>_{\mathrm{Rb0.6}}$ = 5.

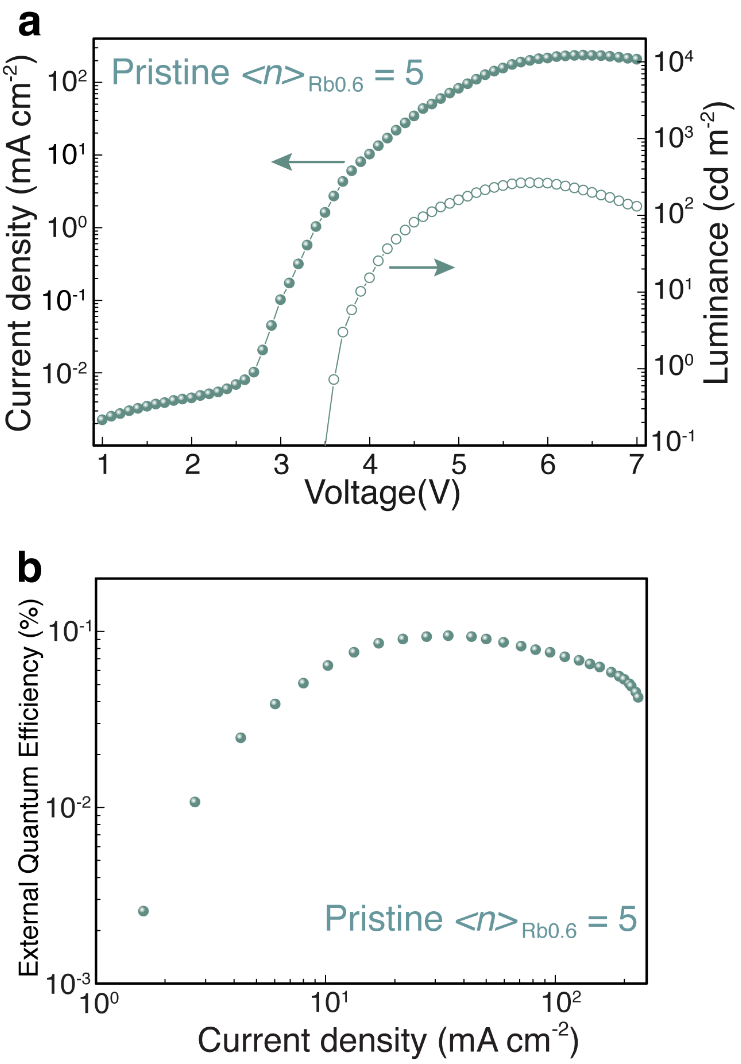

Supplementary Figure 10 | Optoelectronic characteristics of the PeLEDs based on pristine $\langle n \rangle_{Rb0.6}$ = 5. (a) *I-V* and *L-V* curves, and **(b)** EQE-*I* characteristics of pristine $\langle n \rangle_{Rb0.6}$ = 5. It is found that the luminance for pristine $\langle n \rangle_{Rb0.6}$ = 5 quasi-2D perovskite reached 104.6 cd m^{-2}, which is about four-times higher than pristine $\langle n \rangle_{Rb0.6}$ = 3. However, luminance is proportional to the product of current density and EQE. Even though luminance is higher, pristine $\langle n \rangle_{Rb0.6}$ = 5 displayed a fairly large current density indicating charge carrier leakage, which results in an overall low EQE of 0.11%. In addition, the pristine $\langle n \rangle_{Rb0.6}$ = 5 devices display a brightness roll-off at around 6 V, indicating strong non-radiative recombination was taking place.

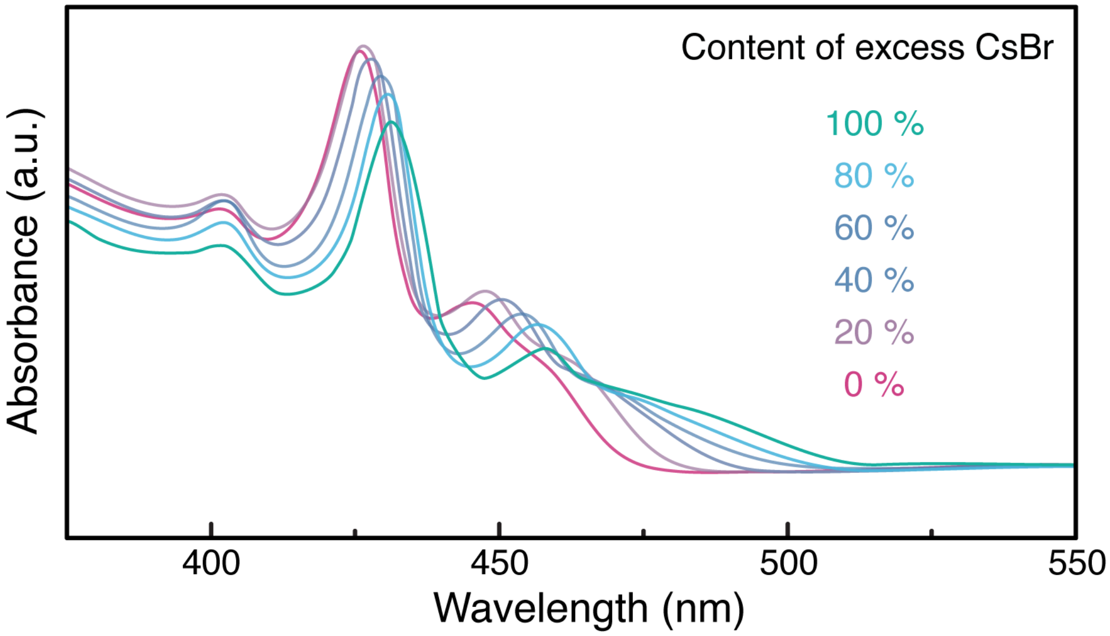

Supplementary Figure 11 | UV-vis absorption spectra of quasi-2D perovskite films. UV-vis absorption spectra of $<n>_{Rb0.6} = 3$ perovskites with different content of excess CsBr. The corresponding CsBr-rich $<n>_{Rb0.6} = 3$ perovskites are also synthesized to explore the different passivation effect between CsBr and RbBr. Unfortunately, cation exchange (Rb^+ replace with Cs^+) is taking place when excessive amount of Cs^+ introduced due to the more reasonable tolerance factor of Cs^+. It means $PEA_2(Rb_{0.2}Cs_{0.8})_2Pb_3Br_{10}$ perovskite is formed when we introduced 100% excess of CsBr into pristine $<n>_{Rb0.6} = 3$ perovskite. Anyhow, excessive amount of CsBr essentially induce a cation exchange leading to a green emitter, which is not interested in this research.

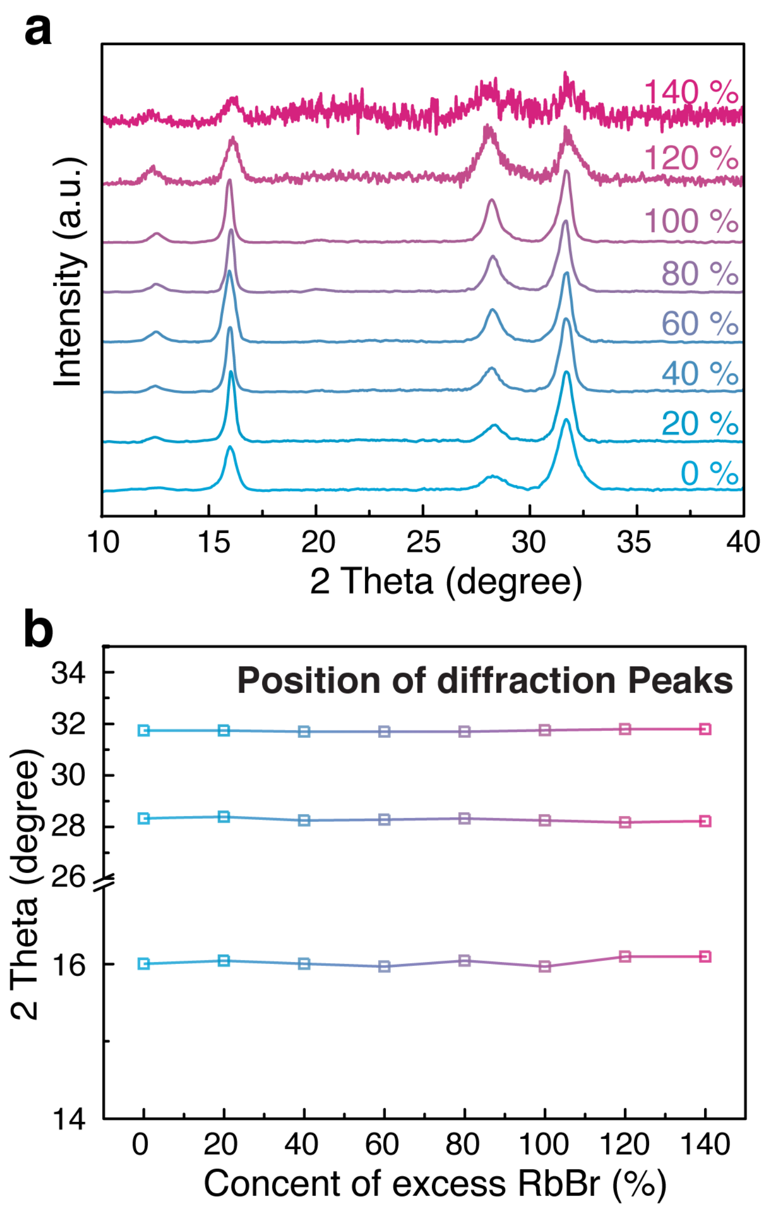

Supplementary Figure 12 | XRD patterns of quasi-2D perovskite films. (a) XRD patterns of $<n>_{\mathrm{Rb0.6}} = 3$ perovskites with different content of excess RbBr, and **(b)** detailed position comparison of the different diffraction peaks.

As shown, three dominant diffraction peaks could be observed, and the diffraction peak at 28° can be indexed to crystallographic plane (2 0 2) of quasi-2D perovskite[6]. With the treatment of excess RbBr, the intensity of (2 0 2) continuous enhanced, indicating that the crystallinity along (2 0 2) plane of RbBr-rich $<n>_{\mathrm{Rb0.6}} = 3$ is much higher than that of pristine analogue. The highly oriented structure reduced the disorder of the crystal and then suppressed the defect state formation[3]. Thus, the passivation

effect of excess RbBr is pluralism, which can also passivate crystal defect by affecting the crystallization process as well as crystal orientation. However, while the content of excess RbBr exceeded 100%, the crystallinity of Rb-Cs alloyed quasi-2D perovskite was significantly reduced, demonstrating that too much RbBr did inhibit the crystallization.

Furthermore, the XRD peaks show no obvious shifted among all the samples, demonstrating the crystal structure as well as the ratio of Rb to Cs in crystals remains unchanged.

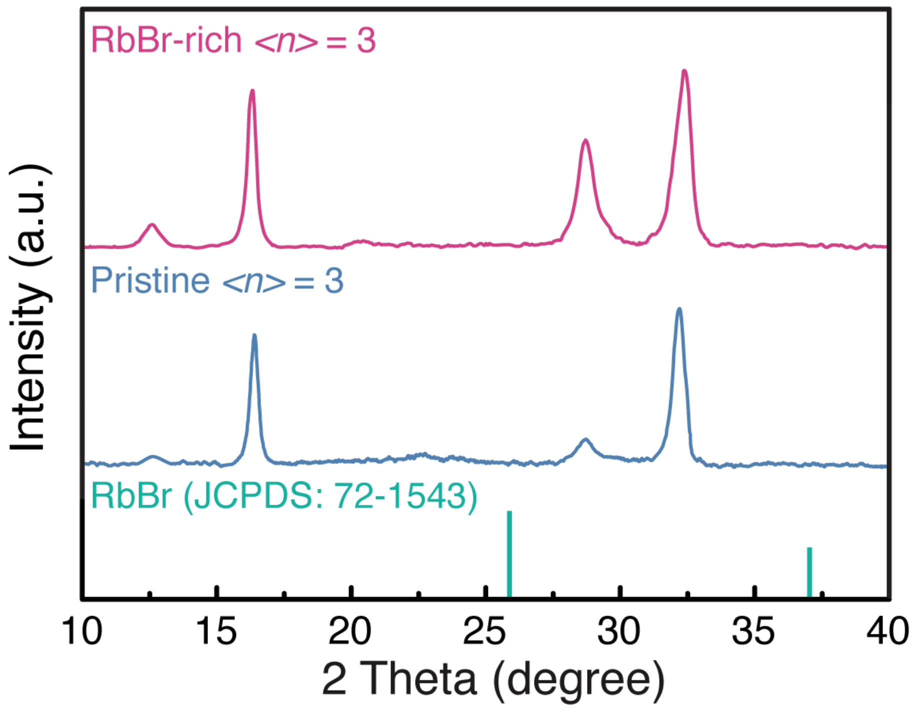

Supplementary Figure 13 | XRD patterns of the perovskite films. XRD characterization demonstrated RbBr-rich $<n>_{Rb0.6} = 3$ perovskite possessed very similar diffraction pattern with its pristine analogue, and no characteristic peak ascribed to RbBr could be detected.

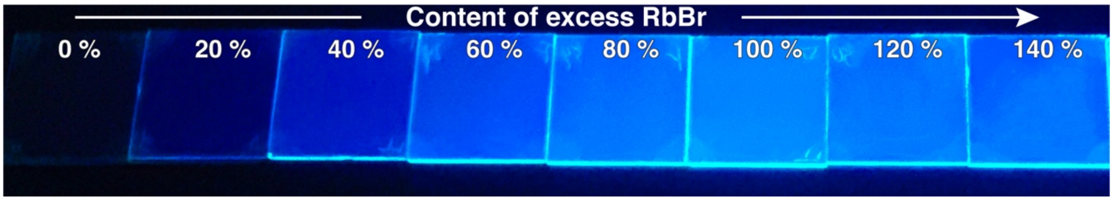

Supplementary Figure 14 | Photoluminescence image of quasi-2D perovskite films. Photoluminescence image of $<n>_{Rb0.6} = 3$ perovskites with different content of excess RbBr under ultraviolet lamp excitation (365 nm). It can be clearly observed that the films become brighter with the increasing content of excess RbBr until 100%, and become weaker with the excess RbBr of 120% and 140%.

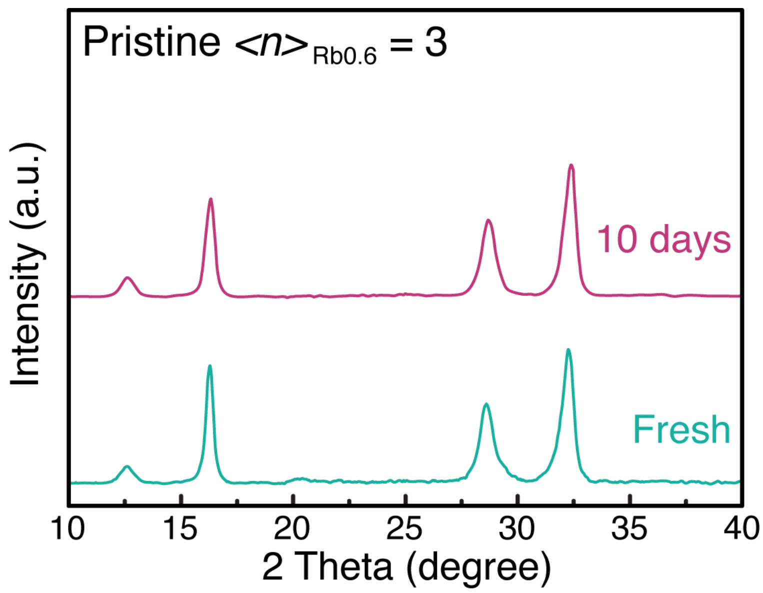

Supplementary Figure 15 | Time evolution of XRD patterns for the perovskite. The XRD pattern of the RbBr-rich $<n>_{Rb0.6} = 3$ perovskite film shows no obvious change even after 10 days, demonstrating the excellent ambient atmosphere stability. The corresponding PLQYs tracking data are shown in Supplementary Figure 30.

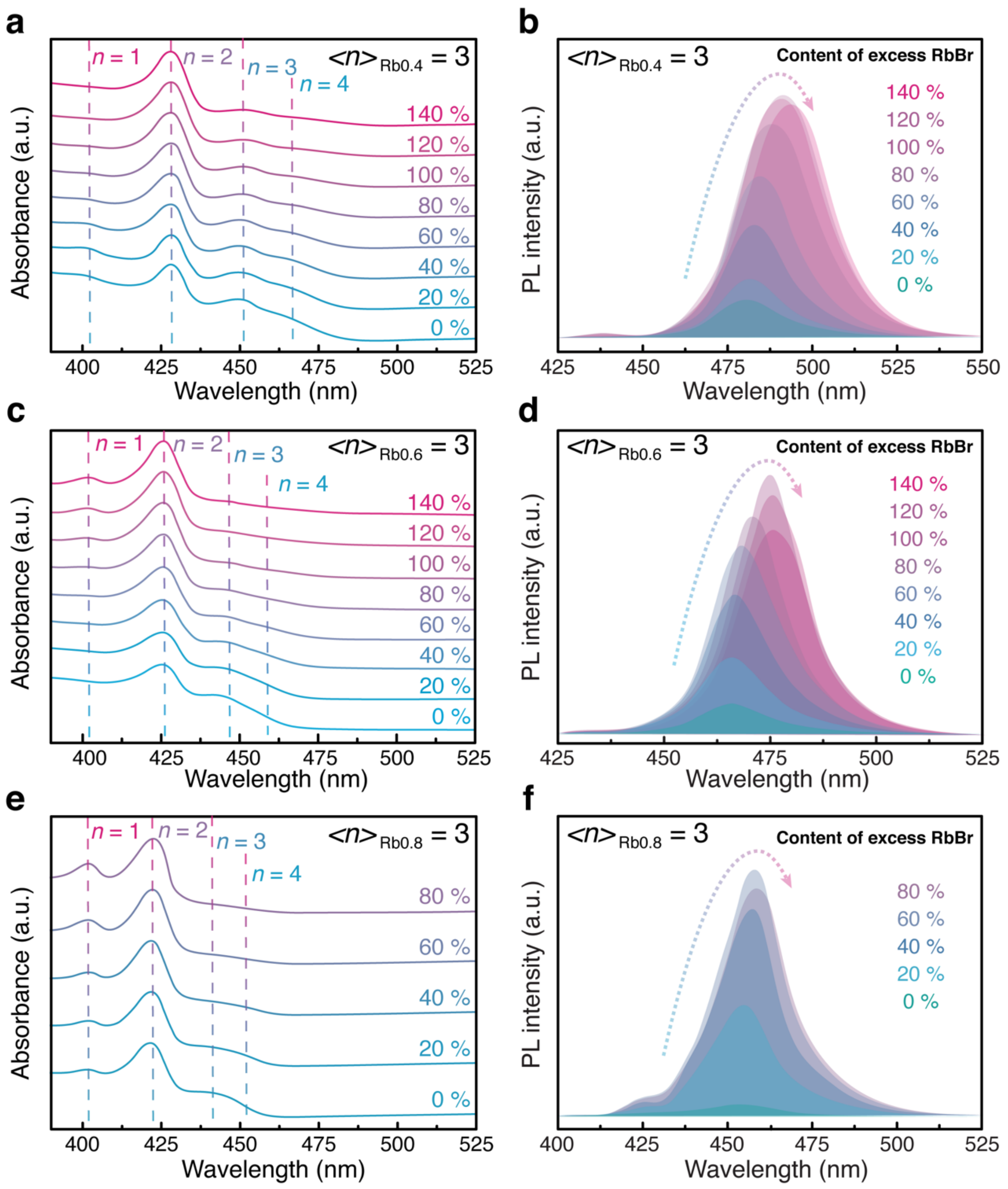

Supplementary Figure 16 | UV-vis absorption and steady-state PL spectra of quasi-2D perovskite with different compositions. (a, c, e) UV-vis absorption and **(b, d, f)** steady-state PL spectra of $<n>_{Rb0.4} = 3$, $<n>_{Rb0.6} = 3$ and $<n>_{Rb0.8} = 3$ perovskites with different content of excess RbBr, respectively. Especially for $<n>_{Rb0.6} = 3$, the PL intensity enhanced with the increasing amount of RbBr until 100% excess (Supplementary Figure 16d). Then, PL intensity started to decline when further induced more RbBr due to the increased trap density (Supplementary Figure 17). Notably, with the different compositions, Rb-Cs alloyed quasi-2D perovskites exhibit a continuous

spectrum at the whole blue-spectra region. The detailed PL emission wavelengths and PLQYs of different compositions are provided in Supplementary Figure 28.

Furthermore, the exciton absorption peaks of $<n>_{\mathrm{Rb0.6}} = 3$ perovskites exhibited no shifting when different amount of excess RbBr induced. Thus, we confirmed the unchanged ratio of Rb to Cs in the Rb-Cs alloyed quasi-2D perovskites, which is also consistent with the results from XRD patterns (Supplementary Figure 12).

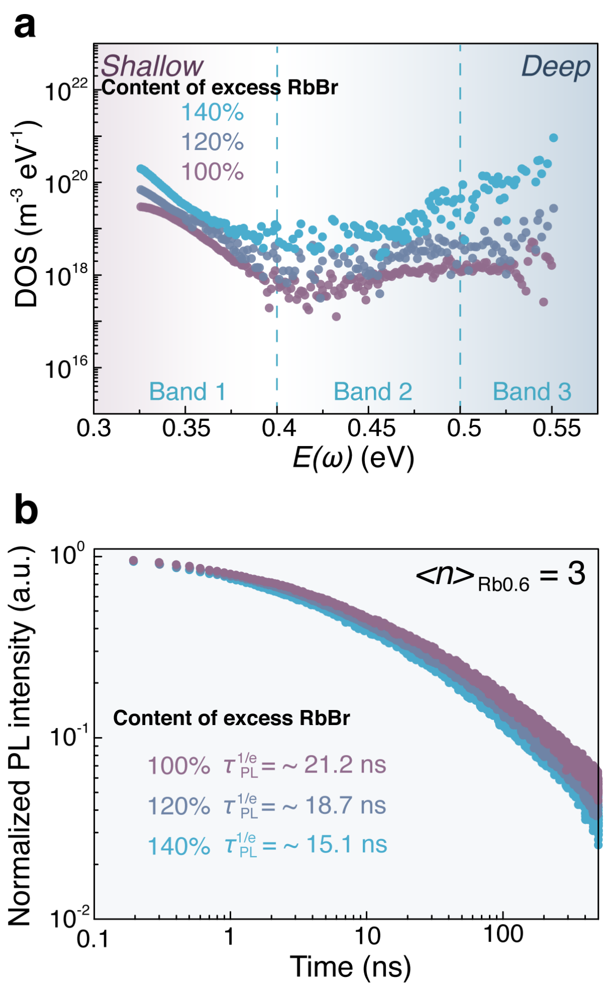

Supplementary Figure 17 | Characteristics of trap density for $<n>_{Rb0.6}$ = 3 perovskites with different content of excess RbBr. (a) TAS analysis of trap density of states (*t*DOS), and **(b)** PL lifetime for $<n>_{Rb0.6}$ = 3 perovskites with different content of excess RbBr. In particular, with the amount of excess RbBr exceed 100%, the perovskite displayed almost one-order of magnitude higher trap density than RbBr-rich $<n>_{Rb0.6}$ = 3, and the PL lifetime is also reduced from around 21.2 to 18.7 and 15.1 ns, indicating a serious non-radiative recombination. Thus, we confirmed that too much RbBr did inhibit the perovskites' crystallization and then formed a large number of defect state, resulting to reduced PLQYs.

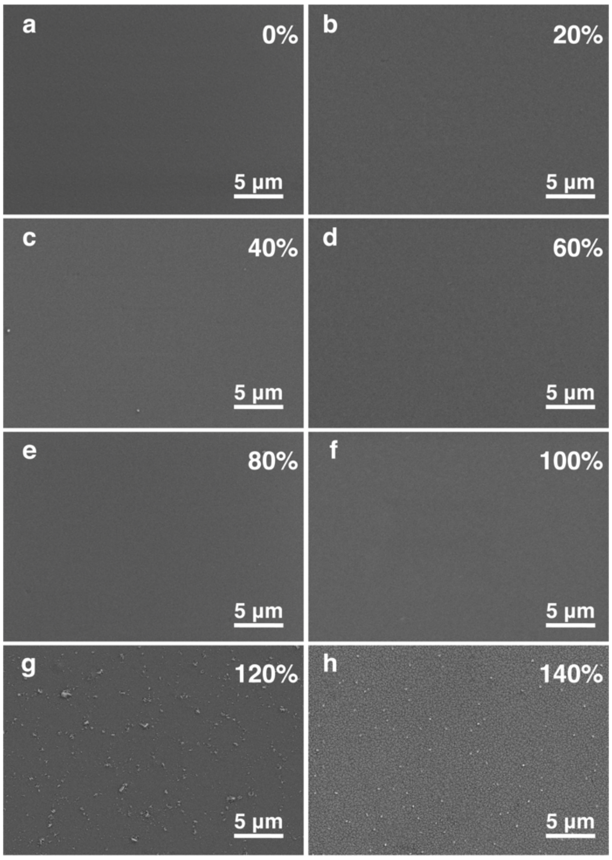

Supplementary Figure 18 | Top-view SEM images of the <*n*>$_{Rb0.6}$ = 3 perovskites with different content of excess RbBr. As shown, with the content of excess RbBr exceed 100%, the immoderate RbBr could induce significant deterioration of the morphology (Supplementary Figure 18g, h)[7].

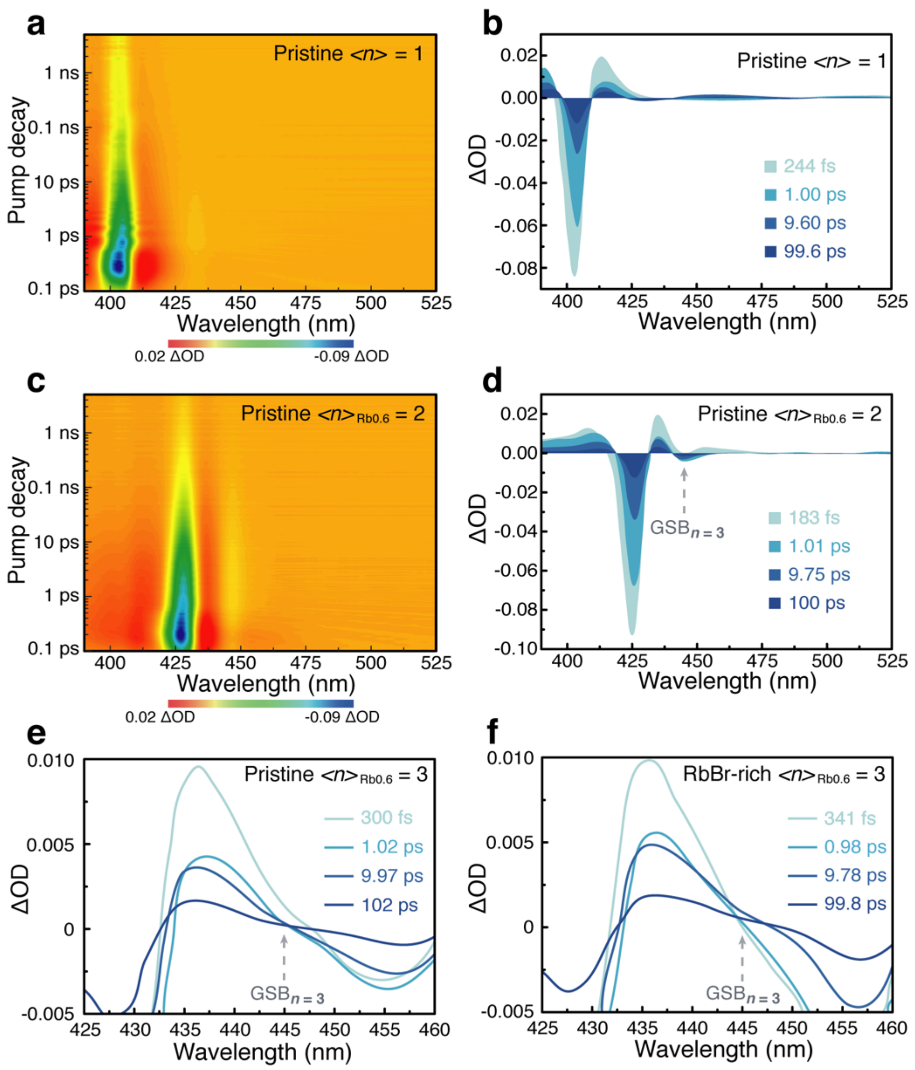

Supplementary Figure 19 | TA measurements for quasi-2D perovskite films. (a, c) Time-wavelength dependent TA color maps and (**b, d)** TA spectra at selected timescales for pristine <*n*> = 1 and pristine <*n*>$_{\text{Rb0.6}}$ = 2 perovskite, respectively. **(e, f)** Enlarged TA spectra at selected timescales for pristine <*n*>$_{\text{Rb0.6}}$ = 3 and RbBr-rich <*n*>$_{\text{Rb0.6}}$ = 3 perovskites, respectively. As shown, it could be confirmed that the GSB of *n* = 3 species is at around 445 nm (Supplementary Note 3).

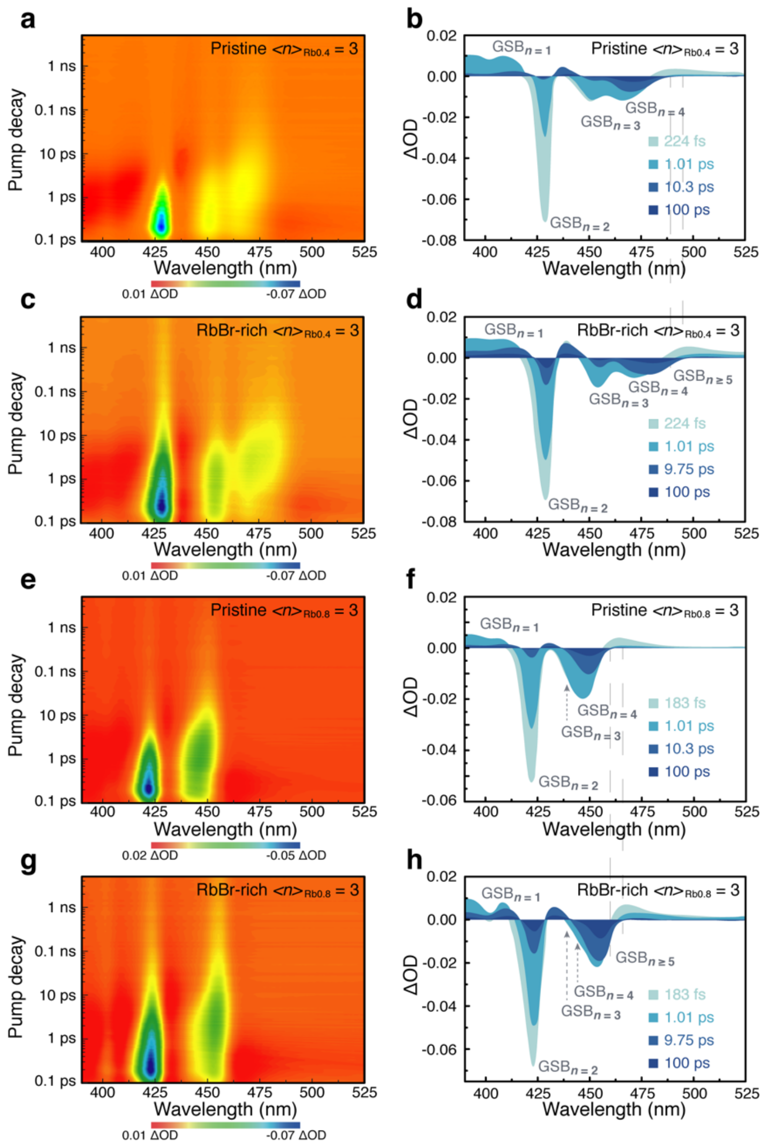

Supplementary Figure 20 | TA measurements for quasi-2D perovskites. (a, c, e, g) Time-wavelength dependent TA color maps and **(b, d, f, h)** TA spectra at selected timescales for pristine <*n*>$_{Rb0.4}$ = 3, RbBr-rich <*n*>$_{Rb0.4}$ = 3, pristine <*n*>$_{Rb0.8}$ = 3 and RbBr-rich <*n*>$_{Rb0.8}$ = 3 perovskites, respectively. As shown, with the increasing ratio of Rb to Cs, we found the GSB peaks for different *n*-value species closely packed together, and then become difficult to be distinguished, which is one of the main reasons for the unappeased of n = 3 GSB signals in <*n*>$_{Rb0.6}$ = 3 perovskites (Supplementary Note 3). Furthermore, with the treatment of excess RbBr, larger *n*-values ($n \geq 5$) species could be found in <*n*>$_{Rb0.4}$ = 3 and <*n*>$_{Rb0.8}$ = 3 perovskites, which is similar with <*n*>$_{Rb0.6}$ = 3 (Fig. 2b, e).

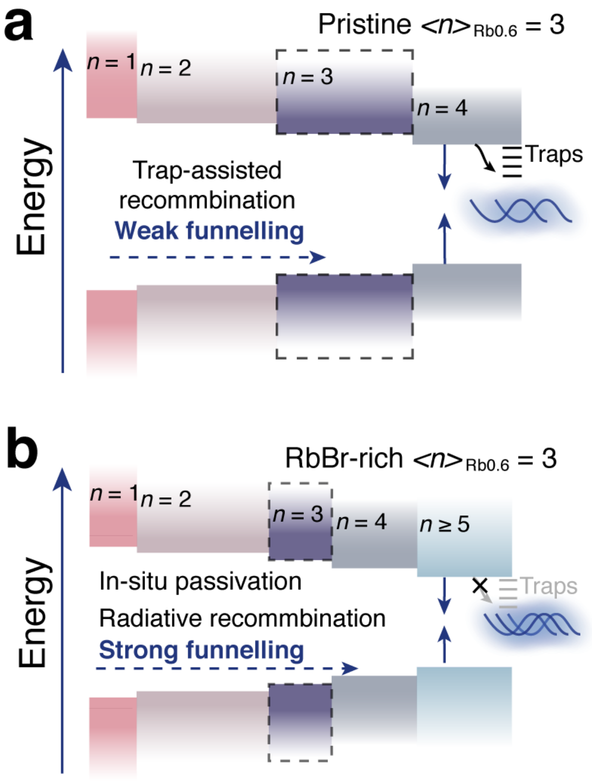

Supplementary Figure 21 | Schematic representation of energy transfer process. Schematic representation of energy transfer process through **(a)** pristine $<n>_{\mathrm{Rb0.6}} = 3$ and **(b)** RbBr-rich $<n>_{\mathrm{Rb0.6}} = 3$ perovskite, respectively.

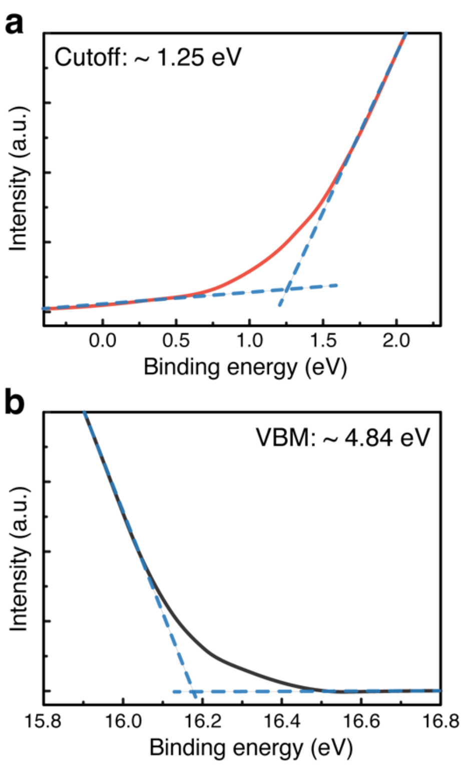

Supplementary Figure 22 | Ultraviolet photoelectron spectroscopy (UPS) of RbBr-rich <*n*>$_{Rb0.6}$ = 3.

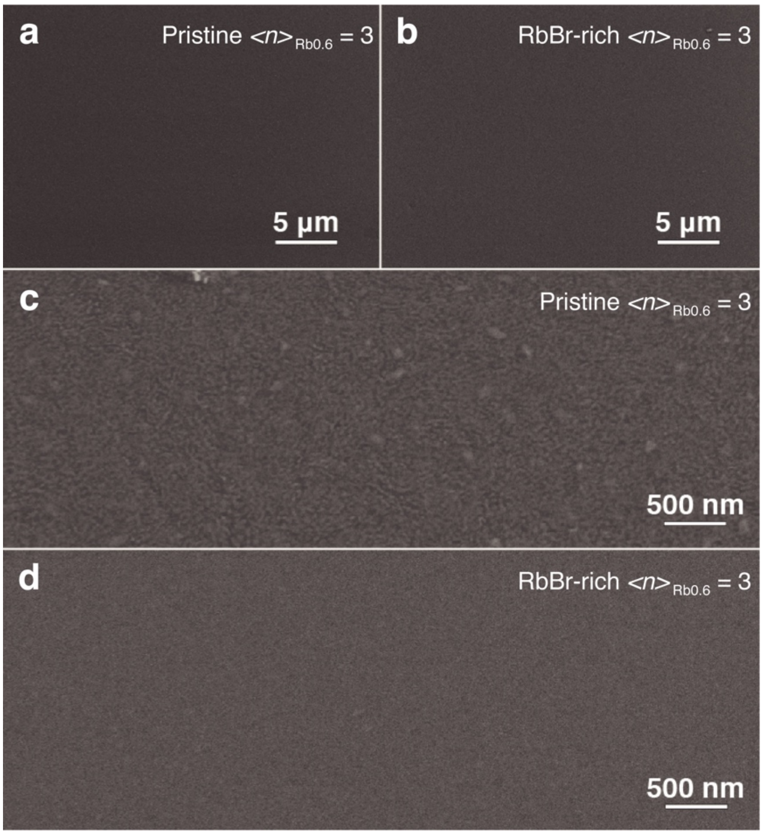

Supplementary Figure 23 | Morphology images of quasi-2D perovskite films. Top-view SEM images of **(a, c)** pristine $<n>_{Rb0.6} = 3$ and **(b, d)** RbBr-rich $<n>_{Rb0.6} = 3$ perovskites. Contrasted with 3D perovskite (Supplementary Figure 5), the surface morphology of the pristine $<n>_{Rb0.6} = 3$ film is significantly improved. Long-chain organic cation of PEA^{+} can act as a surfactant and constrain the growth of perovskite grains during the film forming[8]. Thus, the $<n>_{Rb0.6} = 3$ films exhibit a smaller grain size than 3D counterpart. Surprisingly, the grain size could be further reduced by adding excess RbBr into the system.

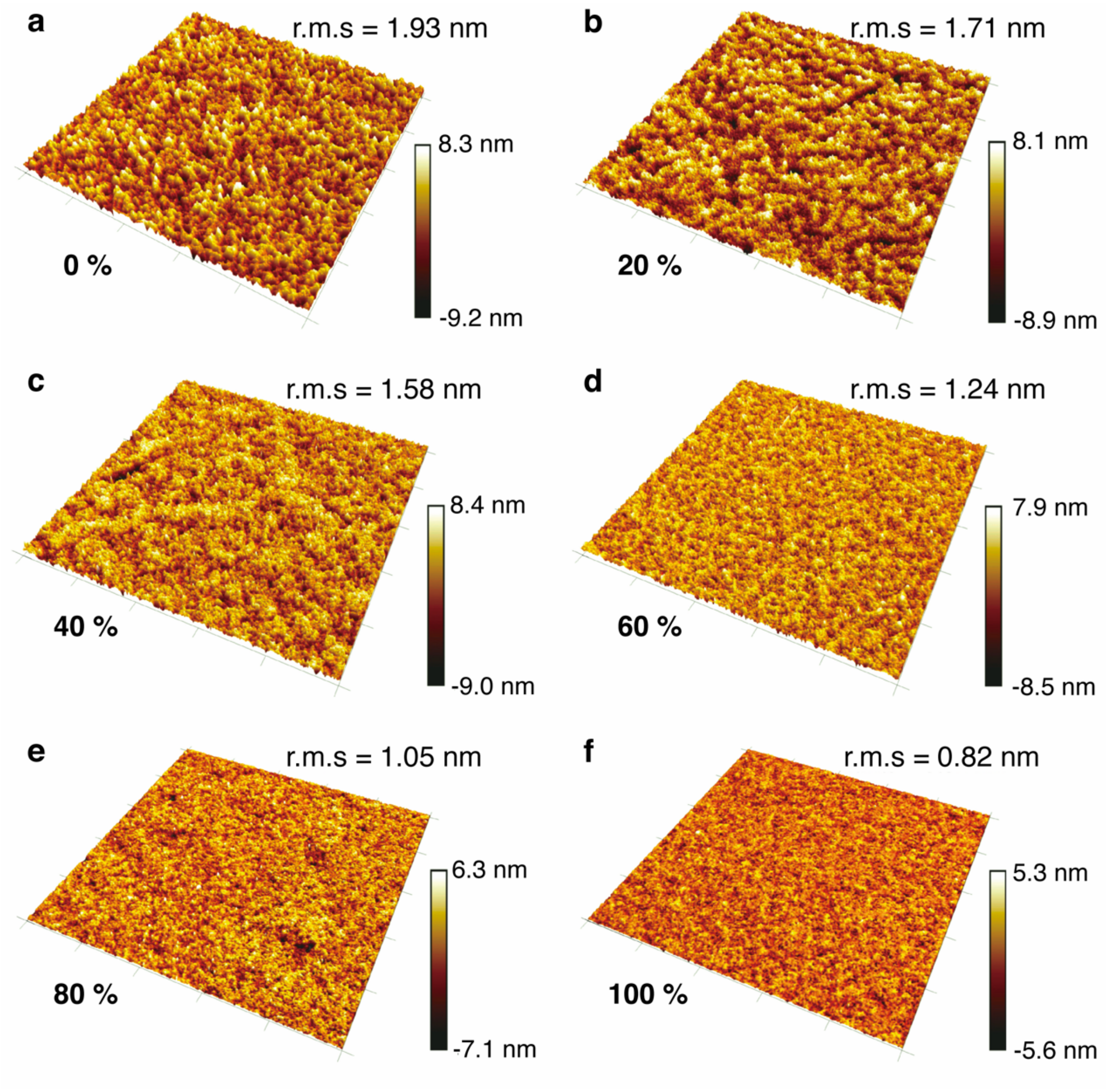

Supplementary Figure 24 | Morphology of quasi-2D perovskite films. Atomic force microscope (AFM) images of $<n>_{Rb0.6}$ = 3 perovskite films coated on PEDOT:PSS layer with different content of excess RbBr. The r.m.s. roughness of the films are marked on the right sides of the images, respectively. The scan of all images is 10 μm × 10 μm. With the content of excess RbBr increasing, the perovskite films become smoother, decreasing the roughness from 1.93 to stupendous 0.82 nm r.m.s. Thus, the addition of excess RbBr in moderation could efficiently prevent the formation of large quasi-2D perovskite grain, and realized high-quality perovskite films.

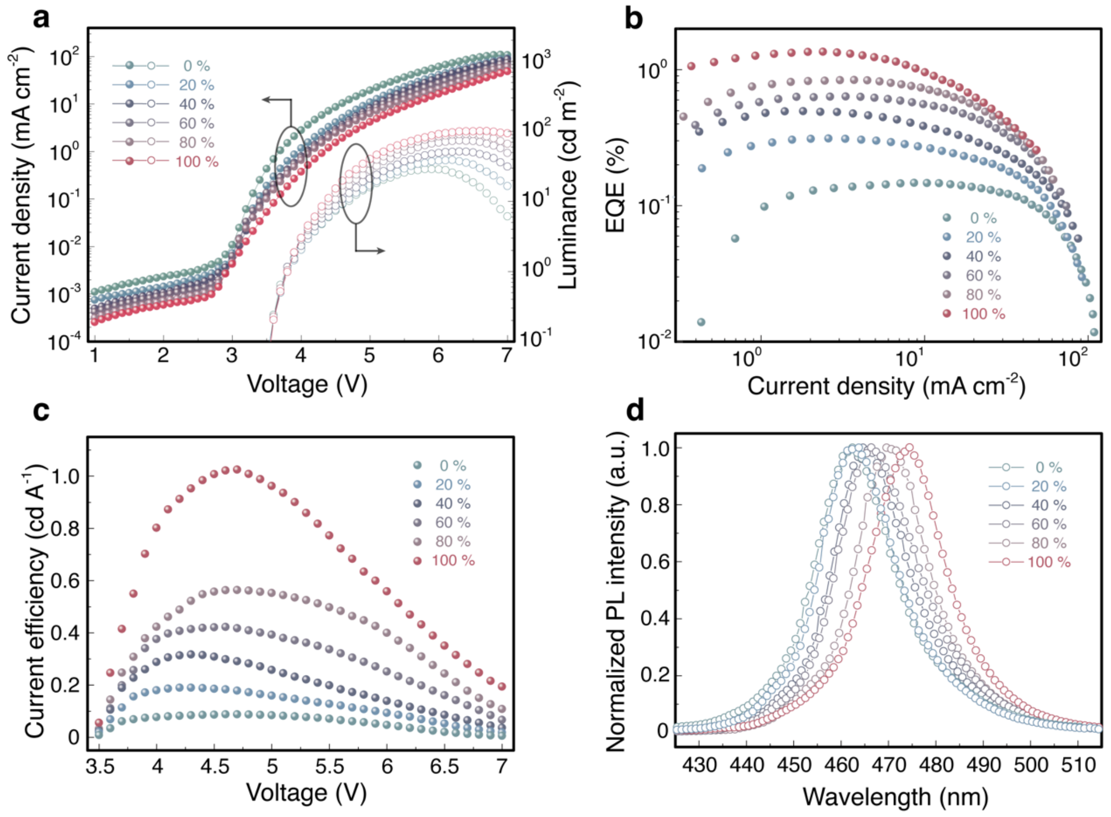

Supplementary Figure 25 | Performance of PeLEDs. (a) *I-V* and *L-V* curves, **(b)** EQE-*I* curves, **(c)** current efficiency-voltage (*CE-V*) curves and **(d)** EL spectra of $<n>_{Rb0.6} = 3$ perovskites with different content of excess RbBr.

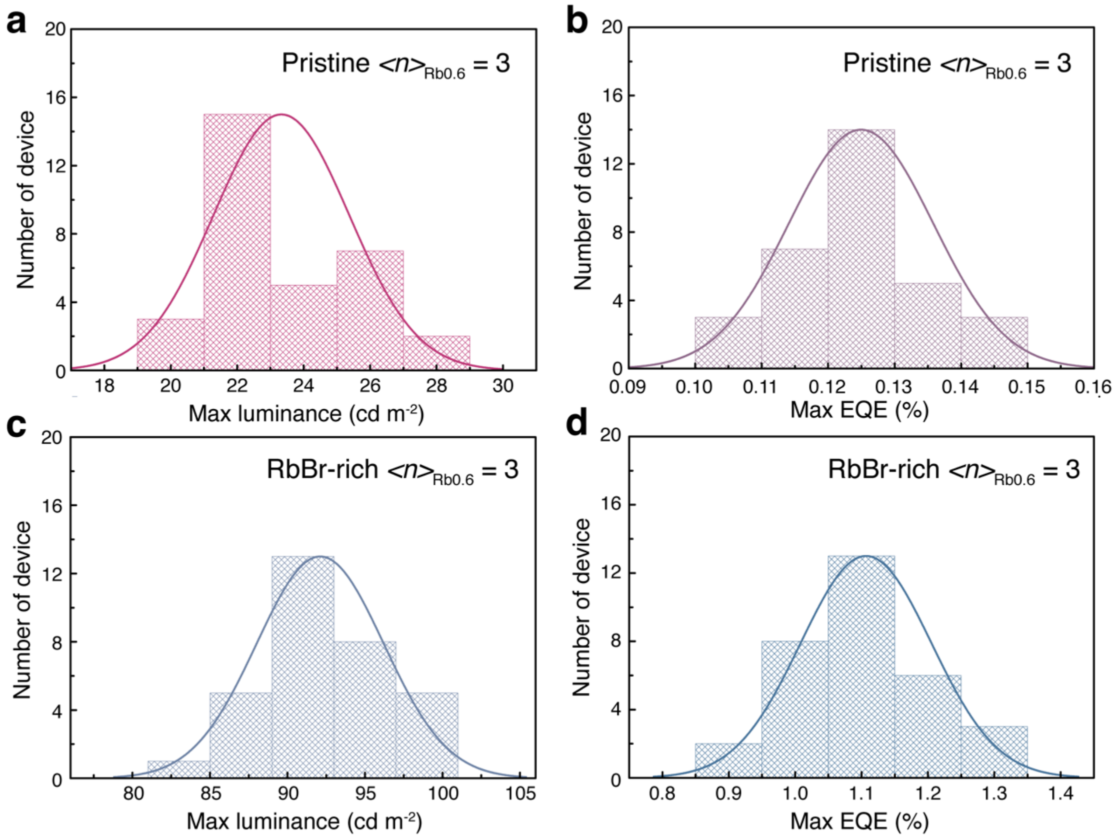

Supplementary Figure 26 | Statistical data for device performance. Histograms of **(a, c)** maximum luminance and **(b, d)** EQEs for pristine $\langle n \rangle_{Rb0.6}$ = 3 and RbBr-rich $\langle n \rangle_{Rb0.6}$ = 3 based devices, respectively. For pristine $\langle n \rangle_{Rb0.6}$ = 3 and RbBr-rich $\langle n \rangle_{Rb0.6}$ = 3 based devices, the average luminance is 23.3 and 92.1 cd m^{-2} with a relative standard deviation of 8.7% and 4.5%, and the average EQE is 0.13% and 1.11% with a relative standard deviation of 8.8% and 8.9%, respectively.

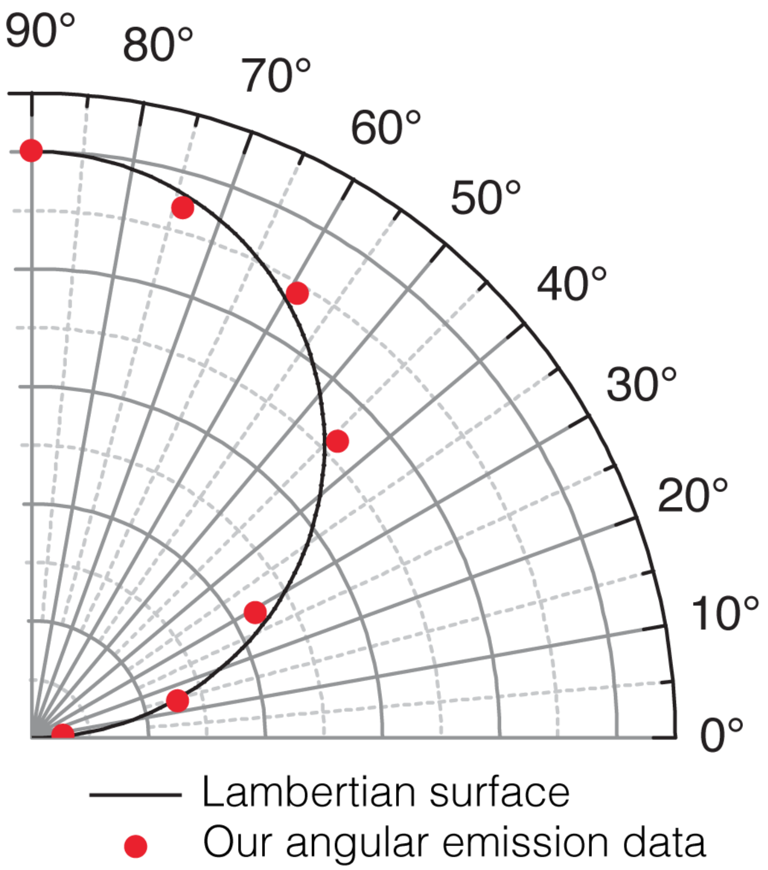

Supplementary Figure 27 | Lambertian characteristic. Angular distribution of radiation intensity for a Lambertian emitter and our RbBr-rich $<n>_{Rb0.6} = 3$ perovskite based PeLEDs.

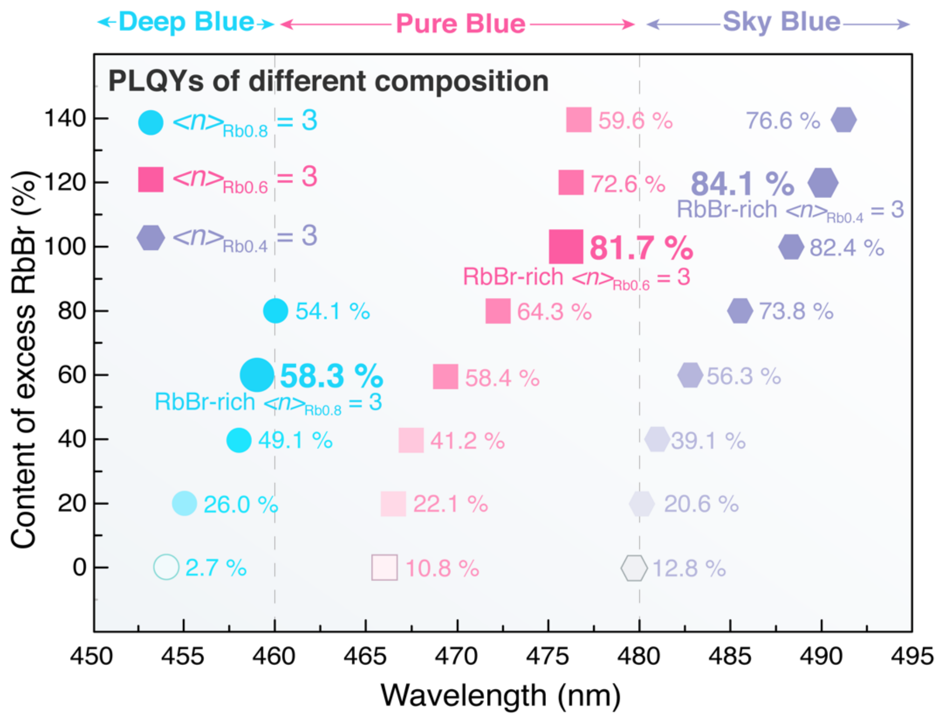

Supplementary Figure 28 | PL emission wavelengths and corresponding PLQYs of the quasi-2D perovskites. PL emission wavelengths and corresponding PLQYs of $<n>_{Rb0.8} = 3$, $<n>_{Rb0.6} = 3$ and $<n>_{Rb0.4} = 3$ perovskites with different content of excess RbBr. As shown, by tuning the composition, we achieved perovskite emitters that covers the entire blue spectral region, from 454 to 492 nm. In particular, RbBr-rich $<n>_{Rb0.8} = 3$ (458 nm, deep-blue) and the RbBr-rich $<n>_{Rb0.4} = 3$ (490 nm, sky-blue) exhibited reasonable PLQYs of 58.3 % and 84.1 %, respectively. Detailed description could be found in Supplementary Note 4.

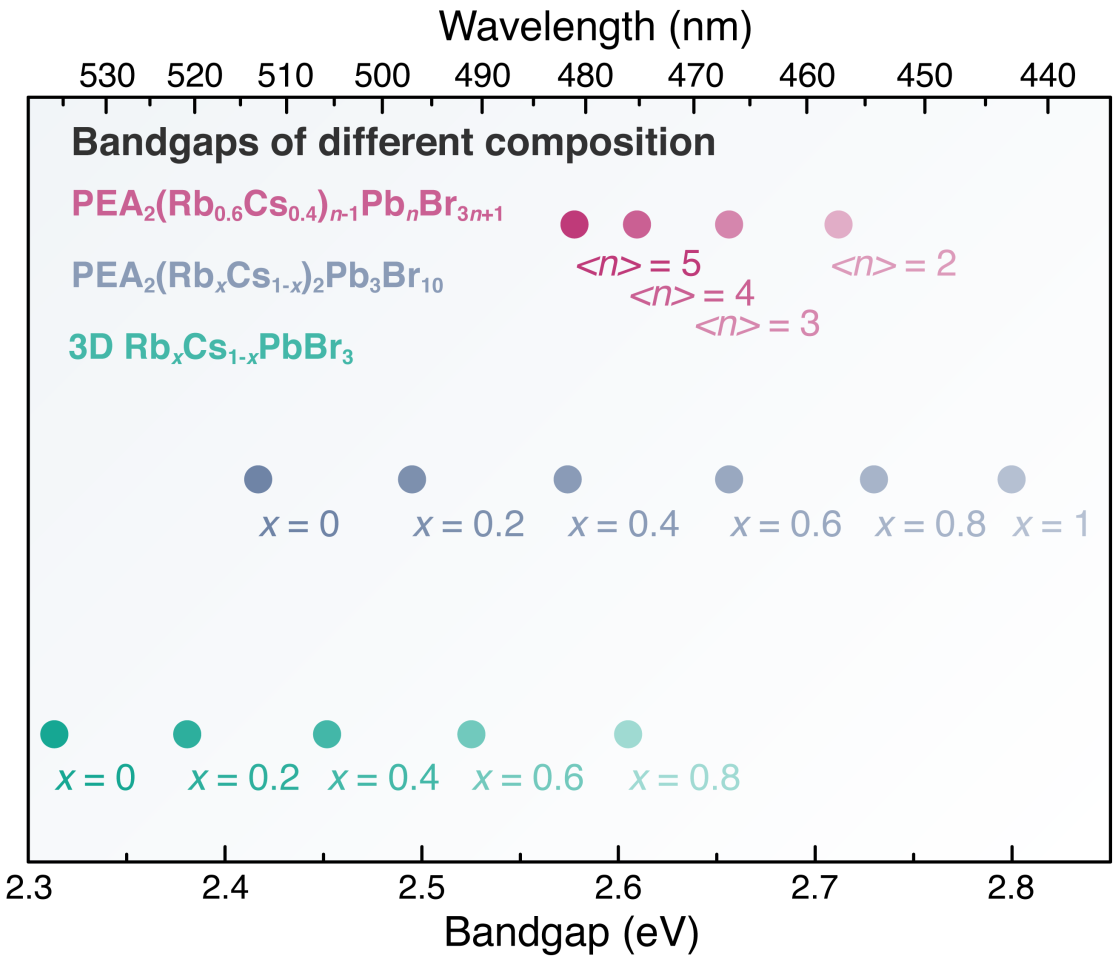

Supplementary Figure 29 | Optical bandgaps of the perovskites. Optical bandgaps of $PEA_2(Rb_{0.6}Cs_{0.4})_{n-1}Pb_nBr_{3n+1}$ ($2 \leq n \leq 5$), $PEA_2(Rb_xCs_{1-x})_2Pb_3Br_{10}$ ($0 \leq x \leq 1$) and $Rb_xCs_{1-x}PbBr_3$ ($0 \leq x \leq 0.8$) calculated from Supplementary Figures 7a, 1b and 2a, respectively. Moreover, the optical bandgap of PEA_2PbBr_4 ($\langle n \rangle = 1$) perovskite is about 3.03 eV, which is not shown in the figure.

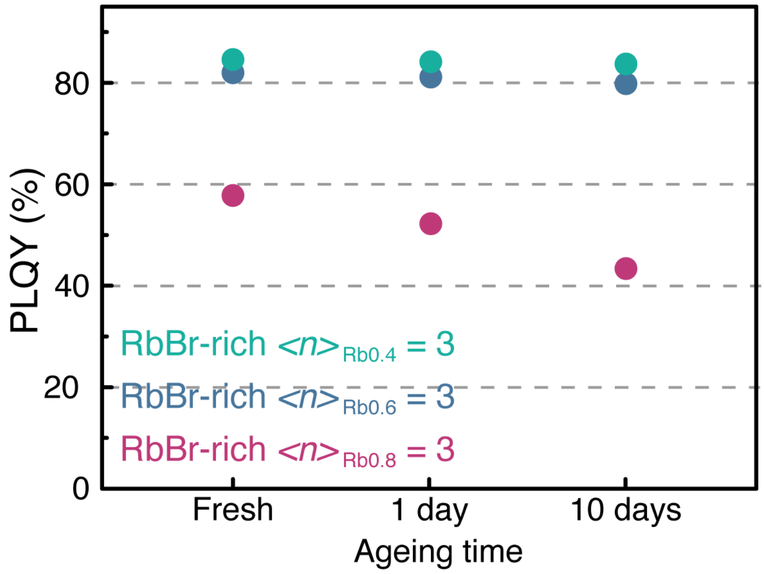

Supplementary Figure 30 | PLQY tracking data for quasi-2D perovskite films. PLQY tracking data for RbBr-rich $<n>_{Rb0.4} = 3$, RbBr-rich $<n>_{Rb0.6} = 3$ and RbBr-rich $<n>_{Rb0.8} = 3$ under ambient condition.

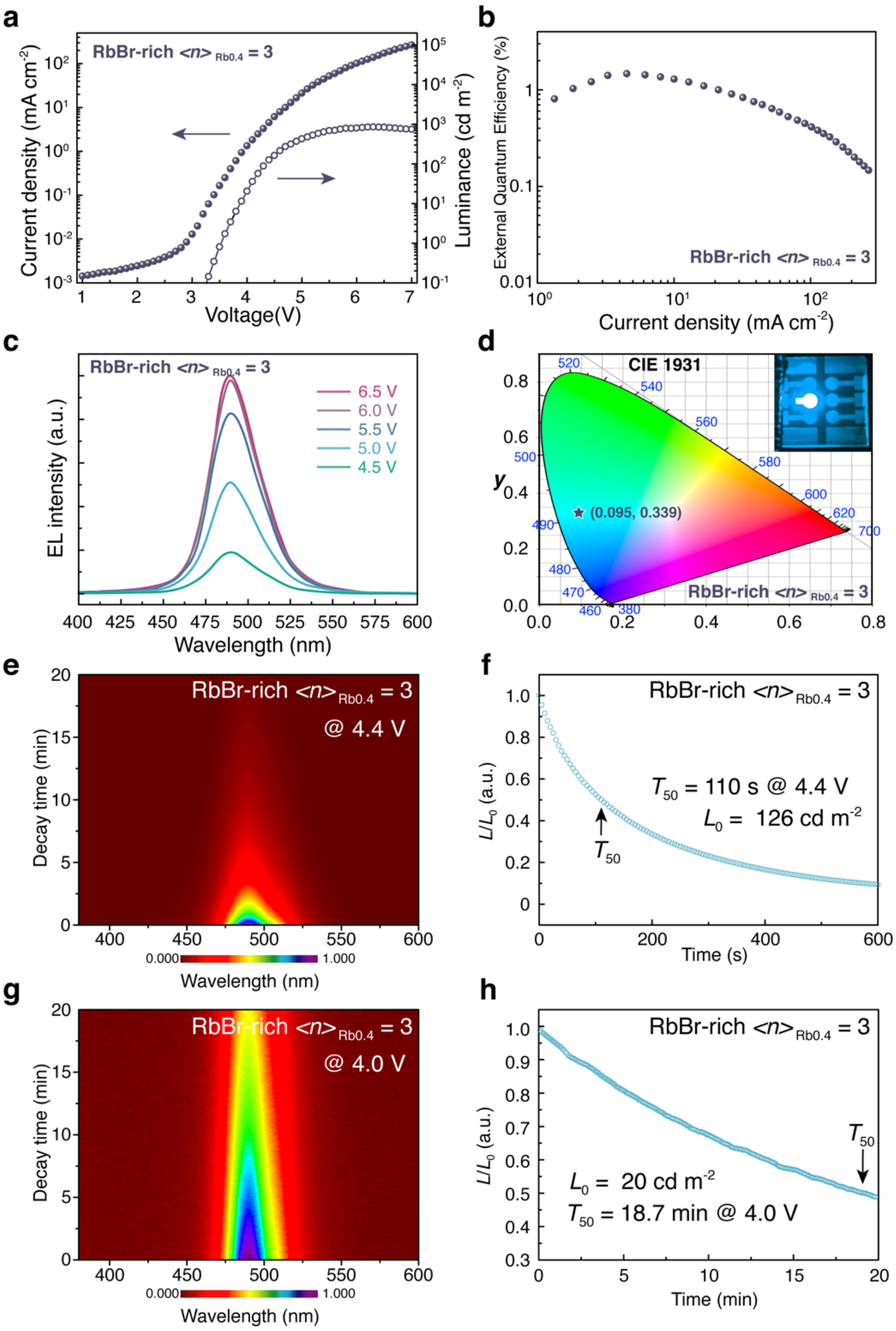

Supplementary Figure 31 | Optoelectronic characteristics of the RbBr-rich <*n*>$_{Rb0.4}$ = 3 based PeLEDs. **(a)** *I-V* and *V-L* curves, and **(b)** EQE-*I* characteristic of RbBr-rich <*n*>$_{Rb0.4}$ = 3 based devices. **(c)** EL spectra of RbBr-rich <*n*>$_{Rb0.4}$ = 3 based devices at different voltage bias. **(d)** The corresponding Commission Internationale de l'Echlaiage (CIE) coordinate of RbBr-rich <*n*>$_{Rb0.4}$ = 3 based devices. EL spectra change under **(e)** max EQE point and **(g)** constant voltage of 4.0 V. Life measurement (T_{50}) of RbBr-rich <*n*>$_{Rb0.4}$ = 3 based devices under **(g)** max EQE point and **(h)** constant voltage of 4.0 V.

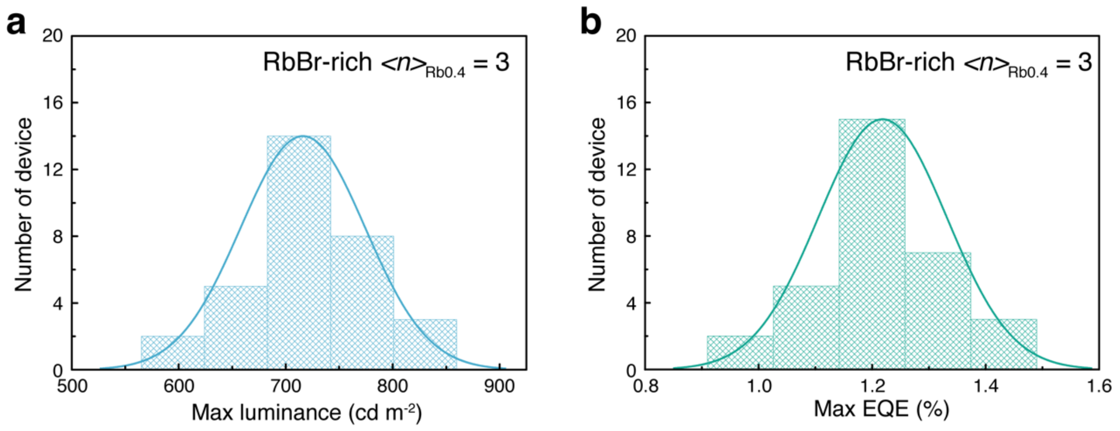

Supplementary Figure 32 | Statistical data for device performance. Histograms of **(a)** maximum luminance and **(b)** EQEs for RbBr-rich $<n>_{Rb0.4} = 3$. The average luminance is 716.1 cd m^{-2} with a relative standard deviation 8.2%, and the average EQE is 1.22% with a relative standard deviation of 9.3%.

Supplementary Table 1 | The perovskite precursor solutions for different compositions. The perovskite precursor solutions of pristine quasi-2D $PEA_2(Rb_{0.6}Cs_{0.4})_{n-1}Pb_nBr_{3n+1}$ and RbBr-rich $<n>_{\mathrm{Rb0.6}} = 3$ perovskites.

Composition	PEABr (mL^{-1})	RbBr (mL^{-1})	CsBr (mL^{-1})	$PbBr_2$ (mL^{-1})
$<n> = 1$	1 mmol (202.1 mg)	-	-	0.5 mmol (183.5 mg)
$<n>_{\mathrm{Rb0.6}} = 2$	0.5 mmol (101.0 mg)	0.15 mmol (24.8 mg)	0.1 mmol (21.3 mg)	0.5 mmol (183.5 mg)
$<n>_{\mathrm{Rb0.6}} = 3$	0.33 mmol (67.4 mg)	0.2 mmol (33.1 mg)	0.13 mmol (27.7 mg)	0.5 mmol (183.5 mg)
$<n>_{\mathrm{Rb0.6}} = 4$	0.25 mmol (50.5 mg)	0.225 mmol (37.2 mg)	0.15 mmol (31.9 mg)	0.5 mmol (183.5 mg)
$<n>_{\mathrm{Rb0.6}} = 5$	0.2 mmol (40.4 mg)	0.24 mmol (39.7 mg)	0.16 mmol (34.0 mg)	0.5 mmol (183.5 mg)
RbBr-rich $<n>_{\mathrm{Rb0.6}} = 3$	0.33 mmol (67.4 mg)	0.4 mmol (66.2 mg)	0.13 mmol (27.7 mg)	0.5 mmol (183.5 mg)

Supplementary Table 2 | Parameters of the XRD patterns. The lattice/unit constants shown in Supplementary Figure 8 are calculated by Bragg diffraction equation: $2d\sin\theta = m\lambda$ (m = 1, 2, 3 ···), where d is the lattice/unit constant, θ is the diffraction angle, m is the order, and λ = 1.54 Å for Kα.

Composition	XRD peak	2θ (°)	θ (°)	m	d (Å)
$Rb_{0.6}Cs_{0.4}PbBr_3$	(1 0 0)	15.58	7.79	1	5.6
	(2 0 0)	31.77	15.89	2	5.7
Pristine $\langle n \rangle = 1$	(0 0 2)	5.29	2.65	1	16.7
	(0 0 4)	10.47	5.23	2	16.8
	(0 0 6)	15.71	7.86	3	16.8
	(0 0 8)	21.06	10.53	4	16.8
	(0 0 10)	26.46	13.23	5	16.8
	(0 0 12)	31.94	15.97	6	16.8
Pristine $\langle n \rangle_{Rb0.6} = 2$	(0 0 4)	7.88	3.94	2	22.4
	(0 0 6)	11.84	5.92	3	22.5
	(0 0 8)	15.77	7.89	4	22.4
	(0 0 10)	19.69	9.85	5	22.5
	(0 0 14)	27.4	13.70	7	22.5
	(0 0 16)	31.77	15.89	8	22.4

Supplementary Table 3 | The corresponding formation time constant (τ_{et}) and first-order decay (τ_1) of the each GSBs shown in Fig. 2 and Supplementary Figure 9. The kinetics are fitted by a multiple-exponential function[9], $\Delta A(t) = a_1\exp(-t/\tau_1) + a_2\exp(-t/\tau_2) + a_3\exp(-t/\tau_3) - c_1\exp(-t/\tau_{et})$, where a_1, a_2, a_3 and c_1 are amplitudes; τ_1, τ_2 and τ_3 are decay time constants and τ_{et} is formation time constant. Due to the complex slow decay process of τ_2 and τ_3, including energy slow transfer process, trap-assisted recombination etc., only τ_1 and τ_{et} are listed in the Table.

Composition	τ	$GSB_{n=1}$ (403 nm)	$GSB_{n=2}$ (427 nm)	$GSB_{n=4}$ (456 nm)	$GSB_{n=5}$ (471 nm)	$GSB_{n>5}$ (488 nm)
Pristine $\langle n\rangle_{Rb0.6} = 3$	τ_1	0.19 ps	0.33 ps	-	-	-
	τ_{et}	0.08 ps	0.10 ps	0.35 ps	-	-
RbBr-rich $\langle n\rangle_{Rb0.6} = 3$	τ_1	0.12 ps	0.31 ps	0.48 ps	-	-
	τ_{et}	0.08 ps	0.09 ps	0.13 ps	0.92 ps	-
Pristine $\langle n\rangle_{Rb0.6} = 5$	τ_1	0.09 ps	0.29 ps	0.41 ps	9.01 ps	-
	τ_{et}	0.08 ps	0.08 ps	0.12 ps	0.45 ps	0.83 ps

Supplementary Table 4 | Comparison of the performance of blue PeLEDs.

Approach	Materials	EL	EQE	FWHM	Reported stability	Ref.
"Mixed-Halide"	$CsPbBr_xCl_{1-x}$	490 nm	1.90%	19 nm	*none*	Ref. 10
	$CsPbBr_{1.5}Cl_{1.5}$	480 nm	0.007%	17 nm	Spectra shift at ~15 s	Ref. 11
	$CsPbBr_xCl_{1-x}$	470 nm	0.07%	20 nm	Spectra shift at ~15 s	Ref. 12
	$CsPbBr_xCl_{1-x}$	469 nm	0.50%	~ 25 nm	T_{50} = 1 s @ 1 mA cm^{-2}	Ref. 13
	$CsPbBr_xCl_{1-x}$	466 nm	2.12%	18 nm	T_{50} = 12 s @ 1 mA cm^{-2}	Ref. 14
"Reduced-dimensional structure"	$OLA_2MA_{n-1}Pb_nBr_{3n+1}$	456 nm	0.024%	18 nm	Decomposed immediately	Ref. 15
	$PEA_2A_{1.5}Pb_{2.5}Br_{8.5}$	490 nm	1.5%	~27 nm	T_{50} = 10 min @ L_0 = 10 cd m^{-2}	Ref. 16
	$POEA_2MA_{n-1}Pb_nBr_{3n+1}$	462 nm	0.06%	~ 24 nm	*none*	Ref. 17
	$BA_2MA_{n-1}Pb_nBr_{3n+1}$	440 nm	0.005%	~ 48 nm	*none*	Ref. 18
New Rb-Cs	$PEA_2(Rb_{0.6}Cs_{0.4})_2Pb_3Br_{10}$	475 nm	1.35%	20 nm	**T_{50} = 14.5 min @ 4.5 V**	**This work**
	$PEA_2(Rb_{0.4}Cs_{0.6})_2Pb_3Br_{10}$	490 nm	1.48%	~30 nm	**T_{50} = 18.7 min @ 4.0 V**	

Supplementary Note 1 | Properties of Rb-Cs alloyed quasi-2D perovskite films with different $<n>$-values. Previous reports have demonstrated that the *n* distributions of quasi-2D perovskites plays important roles on the optical properties of the materials[19,20]. Bearing this in mind, different $<n>$-values perovskites, $PEA_2(Rb_{0.6}Cs_{0.4})_{n-1}Pb_nBr_{3n+1}$ ($1 \leq n \leq 5$), are fabricated to investigate their properties.

Supplementary Figure 7a shows the absorption spectra of the prepared quasi-2D perovskites. As for $<n>_{Rb0.6} \geq 2$, the absorption spectra exhibit several absorption peaks at 403, 427, 445 and 456 nm that can be attributed to characteristic n = 1, 2, 3 and 4 species respectively, demonstrating that the quasi-2D perovskite films consist of varying numbers of perovskite layers rather than a pure phase. XRD patterns were also carried out to confirm the composition (Supplementary Figure 7c, d). As shown, the (0 0 k) diffraction peaks derived from n = 1 and 2 species could be obviously observed in $<n>$ = 1 and $<n>_{Rb0.6}$ = 2 perovskites respectively (Supplementary Figure 8 and Supplementary Table 2), demonstrating the formation of layered structure. Meanwhile, in the low-diffraction-angle region (Supplementary Figure 7c), the (0 0 *k*) diffraction peak could also be found in $<n>_{Rb0.6}$ = 2 and $<n>_{Rb0.6}$ = 3 perovskite respectively, confirmed the quasi-2D structure. With the $<n>$-values increasing, the dielectric quantum confinement effect is reduced, and then the bandgaps become smaller[21]. Consequently, the PL spectra (Supplementary Figure 7b) of quasi-2D perovskite exhibit continues red-shift from around 405 to 480 nm. Meanwhile, the $<n>$ = 1 and $<n>_{Rb0.6}$ = 2 perovskites show extremely low PL intensity and PLQYs (Supplementary Figure 7e) because of the inefficient internal energy transfer[4,22]. Thus, $<n>_{Rb0.6}$ = 3, 4 and 5 seem to be the candidate for further application of PeLEDs. However, the $<n>_{Rb0.6}$ = 5 with a PL emission at around 480 nm also exhibits a relatively low PLQY due to the higher defect density (Supplementary Figure 7f, g). Furthermore, despite of the $<n>_{Rb0.6}$ = 4 exhibits a slightly higher PLQY, the PL spectra is much broad than that of $<n>_{Rb0.6}$ = 3. Thus, after careful screening, in consideration of the color purity, we select $<n>_{Rb0.6}$ = 3 perovskites for further optimization to fabricate LEDs.

Thermal admittance spectroscopy (TAS) technique (Supplementary Figure 7f) and PL lifetime (Supplementary Figure 7g) were also used to investigate the defect density

of $<n>_{\mathrm{Rb0.6}}$ = 3, 4 and 5 perovskites. As shown, the $<n>_{\mathrm{Rb0.6}}$ = 5 perovskite exhibits almost one-order of magnitude higher trap density than $<n>_{\mathrm{Rb0.6}}$ = 3 analogue, due to the larger *n*-values (*n* >> 5) perovskite domain which possess higher defect density. Moreover, though the $<n>_{\mathrm{Rb0.6}}$ = 4 perovskite shows slightly lower trap density than $<n>_{\mathrm{Rb0.6}}$ = 3 analogue, they are still in the same order. Furthermore, the PL lifetime of the perovskites show the same evolutionary trend. The PL lifetime of $<n>_{\mathrm{Rb0.6}}$ = 5 is significantly shorter than $<n>_{\mathrm{Rb0.6}}$ = 3 and 4 perovskites, further demonstrating the serious non-radiative recombination. Thus, the modulation of the *n* distribution should be more precisely.

Supplementary Note 2 | Photocarrier transportation and recombination dynamics in pristine $<n>_{Rb0.6} = 5$. TA spectra for pristine $<n>_{Rb0.6} = 5$ perovskite was recorded to uncover the mechanism for PL spectra broadening (Supplementary Figure 9b). Four distinct GSB peaks were observed instantly, assigned to n = 1, 2, 4 and 5 species. Strikingly, another broad GSB peak at approximately 475 to 500 nm, was gradually emerged with increasing delay time. The broad GSB peak refers to a group of higher-order n-values ($n >> 5$) species, which possessed almost similar bandgap and formed a close-packed flat-energy-landscape domain[19,20]. Energy transfer process was detected from the donor domains (n = 1, 2, 4) to acceptor domains (n = 5 and $n >> 5$), as similar as observed in pristine $<n>_{Rb0.6} = 3$ perovskite. However, an abnormal slow decay component, $\tau_1 = 9.01$ ps, was extracted from the $GSB_{n=5}$ peak. The decay component shall ascribe to the carrier transfer from n = 5 specie to $n >> 5$ domains, which has not been observed in previous pristine $<n>_{Rb0.6} = 3$. Notably, the carrier transfer lifetime ($\tau_1 = 9.01$ ps) is much longer than the carrier injection kinetics for $n >> 5$ domains ($\tau_{et} = 0.83$ ps). The phenomena indicate the electronic states in $n >> 5$ domains are partially saturated, which blocked further carrier transfer into them[23]; the carriers then accumulate into the n = 5 domain and recombine. Accordingly, TRPL and steady-state PL peak arising from both n = 5 and $n >> 5$ domains were observed, result in overall PL peak broadening (Supplementary Figures 7b, 9d). In addition, the PLQY obtained from pristine $<n>_{Rb0.6} = 5$ perovskite was inferior to $<n>_{Rb0.6} = 3$ analogue, deriving from that the larger-n values ($n >> 5$) perovskite domain possess higher defect density because of their decreased stability (Supplementary Figure 7e, f and g).

Therefore, there are two aspects needed to pay attention to obtain ideal blue emitter according to the above analysis. First, we should modulate the n distribution more precisely. That means we need to increase the population of narrower bandgap species to facilitate the energy transfer, but should avoid forming $n >> 5$ species. Secondly, surface passivation needs to be conducted to reduce the intrinsic defect density.

Supplementary Note 3 | Characteristics for GSB peaks of *n* = 3 species in TA measurements. As shown, the GSB peaks of *n* = 1, 2, 3 and 4 species could be easily distinguished in pristine $<n>_{\mathrm{Rb0.4}}$ =3 perovskite (Supplementary Figure 20a). However, with the increasing ratio of Rb to Cs, the bandgap difference between each *n* species become smaller, leading to the resulting GSB peaks closely packed[24]. For instance, in pristine $<n>_{\mathrm{Rb0.8}}$ = 3 perovskite (Supplementary Figure 20e), the *n* = 3 and 4 species could form a broad and continuous bleaching.

We also provided the TA measurement on pristine $<n>_{\mathrm{Rb0.6}}$ = 2, and further confirmed the GSB peak of *n* = 3 GSB peaks is located at around 445 nm (Supplementary Figure 19c, d), which is consistent with the results from UV-vis absorption spectra (Supplementary Figure 16c).

Moreover, due to the complex complicated electronic coupling process among different *n*-value species, a strong excited state absorption (ESA) signal was found at ΔOD > 0 region. The *n* = 3 GSB peaks of pristine $<n>_{\mathrm{Rb0.6}}$ = 3 and RbBr-rich $<n>_{\mathrm{Rb0.6}}$ = 3 perovskites were also hidden by these ESA signals, and became more difficult to be pointed out[25]. However, we still observed some sags at around 445 nm in ESA statuses for pristine $<n>_{\mathrm{Rb0.6}}$ = 3 and RbBr-rich $<n>_{\mathrm{Rb0.6}}$ = 3, which should from *n* = 3 GSB peaks (Supplementary Figure19e, f).

In brief, the unapparent signal of *n* = 3 species GSB peak probably derived from two main reasons. Firstly, the closed packed GSB peaks are too close to be distinguished with the increasing ratio of Rb to Cs. In addition, strong ESA signals at around 440 nm might also significantly influence the signal of *n* = 3 GSB peak.

Supplementary Note 4 | Full spectrum emission perovskites in the entire blue-spectra region. Efficient and spectral stable pure-blue LEDs with λ_{EL} < 480 nm is highly desired for display application[26], and also is as the key issue in our manuscript. Furthermore, in our research, the ratio of Rb to Cs, $\langle n \rangle$-values and amount of RbBr influenced optical properties of the resulting perovskites coherently. Keeping this in mind, by tuning the variable composition, we further provided the full spectrum emission perovskites to meet the various requirements from different industries.

As shown, with the increasing ratio of Rb to Cs in perovskite, the optical bandgap could be significantly blue-shifted (Supplementary Figure 2a, Supplementary Figure 22, Fig. 1b). Thus, tuning the ratio of Rb to Cs could be an efficient method to regulate the emission wavelength. However, similar with $\langle n \rangle_{\mathrm{Rb0.6}}$ perovskite, the pristine $\langle n \rangle_{\mathrm{Rb0.8}} = 3$ and pristine $\langle n \rangle_{\mathrm{Rb0.4}} = 3$ showed low PLQYs of 2.7% and 12.8%, respectively. As studied in our research, excess RbBr plays dual role in improving the Rb-Cs alloyed quasi-2D perovskites' property. As expected, the PLQYs of $\langle n \rangle_{\mathrm{Rb0.8}} = 3$ and $\langle n \rangle_{\mathrm{Rb0.4}} = 3$ could be largely increased to 58.3% and 84.1% after the treatment of excess RbBr. Furthermore, with the different content of excess RbBr, the spectra exhibit continuous red-shift and then covers the emission region from 454 (deep-blue) to 492 nm (sky-blue) (Supplementary Figure 16, Supplementary Fig 20). In particular, RbBr-rich $\langle n \rangle_{\mathrm{Rb0.8}} = 3$ (458 nm, deep-blue) and the RbBr-rich $\langle n \rangle_{\mathrm{Rb0.4}} = 3$ (490 nm, sky-blue) exhibited reasonable PLQYs of 58.3 % and 84.1 %, respectively.

In summary, we showed the 'A-site' cation engineering strategy to be a versatile method to stabilize the perovskite and regulate the emission properties. This strategy now was confirmed to be able to generate efficient perovskite blue emitters and LEDs in the entire blue spectral region. Thus, we believe that the strategy in our manuscript could open an avenue for PeLED community.

Supplementary Note 5 | Optoelectronic characteristics and spectral stability of the sky-blue PeLEDs based on RbBr-rich $<n>_{Rb0.4}$ = 3. We also fabricated a spectra-stable sky-blue PeLEDs following the same strategy without systematic optimization to demonstrate the versatile application of this technique. The *J-V-L* curves were tested and shown in Supplementary Figure 31a. The turn-on voltage (V_{on}) is slightly reduced compared with RbBr-rich $<n>_{Rb0.6}$ = 3 probably due to the smaller bandgap and injection barrier. With the bias voltage increases, the EL peak at 490 nm remained stable (Supplementary Figure 31c), showing a maximum luminance of 854.3 cd m^{-2} and a high color purity (FWHM is around 30 nm) with Commission Internationale de l'Eclairage (CIE) color coordinate of (0.095, 0.339). Meanwhile, the devices exhibit a peak EQE of 1.48% at 4.4 V (Supplementary Figure 31b). The statistical date for the devices is also shown in Supplementary Figure 32.

Furthermore, we also record the spectral stability of the PeLEDs under continuous voltages. Firstly, the sky-blue device was operated at max EQE point (4.4 V) with a high L_0 of 126 cd m^{-2}. The EL spectra changed negligibly under 20 min and shows a striking T_{50} of 110 s. In order to compare the T_{50} of the device with RbBr-rich $<n>_{Rb0.6}$ = 3 and previous research, the life measurement was also carried out with L_0 = 20 cd m^{-2} at 4.0 V. As shown in Supplementary Figure 31g, h, the T_{50} of the device reached as long as 18.7 min, representing one of the most stable sky-blue PeLEDs.

The higher EQE as well as T_{50} of RbBr-rich $<n>_{Rb0.4}$ = 3 compared with RbBr-rich $<n>_{Rb0.6}$ = 3 could be attributed to two main reasons. Firstly, the lower ratio of Rb to Cs in perovskite could increase the crystal stability (Supplementary Figure 30), resulting in a higher PLQY. Moreover, the narrower bandgap of RbBr-rich $<n>_{Rb0.4}$ = 3 could enhance the injection of carriers and reduce the Joule heating, which could seriously destroy the organic injection layers[27].

Supplementary Method 1 | The UPS measurements for perovskite films. For the UPS measurements as shown in Supplementary Figure 22, the He I (hv = 21.22 eV) emission source was employed and the helium pressure in the chamber during analysis was about 2 × 10^{-8} mbar. The perovskite film samples over the ITO layer were connected with an Au standard sample, and the E_{Fermi} is 21.02 eV. The work function W_F of the measured sample can be calculated from following equation[28]: $hv - W_F = E_{Fermi} - E_{cutoff}$, where the E_{cutoff} is the steep edge position of the test film in the UPS spectrum. The valence band (E_V) and conduction band (E_C) of the film sample can be calculated by the $E_V = W_F + VBM$ and $E_C = W_F + VBM - E_g$ respectively, where the VBM is the valence band maximum and the E_g is the bandgap calculated by UV-vis measurements. As calculated, the valence band (E_V) and conduction band (E_C) of RbBr-rich $\langle n \rangle_{Rb0.6} = 3$ perovskite film is 6.29 eV and 3.68 eV, respectively.

Supplementary References

1. Linaburg, M. R., McClure, E. T., Majher, J. D. & Woodward, P. M. $Cs_{1-x}Rb_xPbCl_3$ and $Cs_{1-x}Rb_xPbBr_3$ solid solutions: understanding octahedral tilting in lead halide perovskites. *Chem. Mater.* **29**, 3507-3514 (2017).
2. Liu, M. *et al.* Hybrid organic-inorganic inks flatten the energy landscape in colloidal quantum dot solids. *Nat. Mater.* **16**, 258-263 (2017).
3. Wang, H. *et al.* Trifluoroacetate induced small-grained $CsPbBr_3$ perovskite films result in efficient and stable light-emitting devices. *Nat. Commun.* **10**, 655 (2019).
4. Yang, X. *et al.* Efficient green light-emitting diodes based on quasi-two-dimensional composition and phase engineered perovskite with surface passivation. *Nat. Commun.* **9**, 570 (2018).
5. Kitazawa, N., & Watanabe, Y. Optical properties of natural quantum-well compounds $(C_6H_5\text{-}C_nH_{2n}\text{-}NH_3)_2PbBr_4$ (n = 1-4). *J. Phys. Chem. Solids* **71**, 797-802 (2010).
6. Tsai, H. *et al.* High-efficiency two-dimensional Ruddlesden-Popper perovskite solar cells. *Nature* **536**, 312 (2016).
7. Yang, M. *et al.* Perovskite ink with wide processing window for scalable high-efficiency solar cells. *Nat. Energy* **2**, 17038 (2017).
8. Xiao, Z. *et al.* Efficient perovskite light-emitting diodes featuring nanometre-sized crystallites. *Nat. Photonics* **11**, 108-115 (2017).
9. Liu, J., Leng, J., Wu, K., Zhang, J. & Jin, S. Observation of internal photoinduced electron and hole separation in hybrid two-dimentional perovskite films. *J. Am. Chem. Soc.* **139**, 1432-1435 (2017).
10. Pan, J. *et al.* Highly efficient perovskite-quantum-dot light-emitting diodes by surface engineering. *Adv. Mater.* **28**, 8718-8725 (2016).
11. Li, G. *et al.* Highly efficient perovskite nanocrystal light-emitting diodes enabled by a universal crosslinking method. *Adv. Mater.* **28**, 3528-3534 (2016).
12. Yao, E. P. *et al.* High-brightness blue and white LEDs based on inorganic perovskite nanocrystals and their composites. *Adv. Mater.* **29**, 1606859 (2017).

13. Gangishetty, M. K., Hou, S., Quan, Q. & Congreve, D. N. Reducing architecture limitations for efficient blue perovskite light-emitting diodes. *Adv. Mater.* **30**, 1706226 (2018).
14. Hou, S., Gangishetty, M. K., Quan, Q. & Congreve, D. N. Efficient blue and white perovskite light-emitting diodes via manganese doping. *Joule* **2**, 2421-2433 (2018).
15. Kumar, S. *et al.* Efficient blue electroluminescence using quantum-confined two-dimensional perovskites. *ACS Nano* **10**, 9720-9729 (2016).
16. Xing, J. *et al.* Color-stable highly luminescent sky-blue perovskite light-emitting diodes. *Nat. Commun.* **9**, 3541 (2018).
17. Chen, Z. *et al.* High-performance color-tunable perovskite light emitting devices through structural modulation from bulk to layered film. *Adv. Mater.* **29**, 1603157 (2017).
18. Congreve, D. N. *et al.* Tunable light-emitting diodes utilizing quantum-confined layered perovskite emitters. *ACS Photonics* **4**, 476-481 (2017).
19. Quan, L. N. *et al.* Tailoring the energy landscape in quasi-2D halide perovskites enables efficient green-light emission. *Nano Lett.* **17**, 3701-3709 (2017).
20. Zou, W. *et al.* Minimising efficiency roll-off in high-brightness perovskite light-emitting diodes. *Nat. Commun.* **9**, 608 (2018).
21. Jiang, Y. *et al*. Reduced-dimensional α-$CsPbX_3$ perovskites for efficient and stable photovoltaics. *Joule* **2**, 1356-1368 (2018).
22. Yuan, M. *et al.* Perovskite energy funnels for efficient light-emitting diodes. *Nat. Nanotech.* **11**, 872 (2016).
23. Xing, G. *et al.* Transcending the slow bimolecular recombination in lead-halide perovskites for electroluminescence. *Nat. Commun.* **8**, 14558 (2017).
24. Li, M. *et al.* Amplified spontaneous emission based on 2D Ruddlesden–Popper perovskites. *Adv. Funct. Mater.* **28**, 1707006 (2018).
25. Shang, Q. *et al*. Unveiling structurally engineered carrier dynamics in hybrid quasi-two-dimensional perovskite thin films toward controllable emission. *J. Phys. Chem. Lett.* **8**, 4431-4438 (2017).

26. Wu, Y., Li, X., & Zeng, H. Highly luminescent and stable halide perovskite nanocrystals. *ACS Energy Lett.* **4**, 673-681 (2019).

27. Qasim, K. *et al*. Solution-processed extremely efficient multicolor perovskite light-emitting diodes utilizing doped electron transport layer. *Adv. Funct. Mater*. **27**, 1606874 (2017).

28. Zhang, L. *et al.* Ultra-bright and highly efficient inorganic based perovskite light-emitting diodes. *Nat. Commun.* **8**, 15640 (2017).
